# Incidence determinants and serological correlates of reactive symptoms following SARS-CoV-2 vaccination

**DOI:** 10.1038/s41541-023-00614-0

**Published:** 2023-02-25

**Authors:** Hayley Holt, David A. Jolliffe, Mohammad Talaei, Sian Faustini, Giulia Vivaldi, Matthew Greenig, Alex G. Richter, Ronan A. Lyons, Christopher J. Griffiths, Frank Kee, Aziz Sheikh, Gwyneth A. Davies, Seif O. Shaheen, Adrian R. Martineau

**Affiliations:** 1 Wolfson Institute of Population Health, Barts and The London School of Medicine and Dentistry, Queen Mary University of London, London, UK; 2 Blizard Institute, Barts and The London School of Medicine and Dentistry, Queen Mary University of London, London, UK; 3 Asthma UK Centre for Applied Research, Queen Mary University of London, London, UK; 4 Institute of Immunology and Immunotherapy, College of Medical and Dental Sciences, University of Birmingham, Birmingham, UK; 5 Population Data Science, Swansea University Medical School, Singleton Park, Swansea, UK; 6 Centre for Public Health Research (NI), Queen’s University Belfast, Belfast, UK; 7 Usher Institute, University of Edinburgh, Edinburgh, UK

**Keywords:** Risk factors, Fever, RNA vaccines

## Abstract

Prospective population-based studies investigating associations between reactive symptoms following SARS-CoV-2 vaccination and serologic responses to vaccination are lacking. We therefore conducted a study in 9003 adults from the UK general population receiving SARS-CoV-2 vaccines as part of the national vaccination programme. Titres of combined IgG/IgA/IgM responses to SARS-CoV-2 spike (S) glycoprotein were determined in eluates of dried blood spots collected from all participants before and after vaccination. 4262 (47.3%) participants experienced systemic reactive symptoms after a first vaccine dose. Factors associating with lower risk of such symptoms included older age (aOR per additional 10 years of age 0.85, 95% CI: 0.81–0.90), male vs. female sex (0.59, 0.53–0.65) and receipt of an mRNA vaccine vs. ChAdOx1 nCoV-19 (0.29, 0.26–0.32 for BNT162b2; 0.06, 0.01–0.26 for mRNA-1273). Higher risk of such symptoms was associated with SARS-CoV-2 seropositivity and COVID-19 symptoms prior to vaccination (2.23, 1.78–2.81), but not with SARS-CoV-2 seropositivity in the absence of COVID-19 symptoms (0.94, 0.81–1.09). Presence vs. absence of self-reported anxiety or depression at enrolment associated with higher risk of such symptoms (1.24, 1.12–1.39). Post-vaccination anti-S titres were higher among participants who experienced reactive symptoms after vaccination vs. those who did not (*P* < 0.001). We conclude that factors influencing risk of systemic symptoms after SARS-CoV-2 vaccination include demographic characteristics, pre-vaccination SARS-CoV-2 serostatus and vaccine type. Participants experiencing reactive symptoms following SARS-CoV-2 vaccination had higher post-vaccination titres of IgG/A/M anti-S antibodies. Improved public understanding of the frequency of reactogenic symptoms and their positive association with vaccine immunogenicity could potentially increase vaccine uptake.

## Introduction

The COVID-19 pandemic is the greatest threat in a generation to global health, having caused more than 6.1 million deaths to date^1^. SARS-CoV-2 vaccination represents the mainstay of disease control, but uptake is suboptimal, largely owing to vaccine hesitancy^2^. Fear of experiencing post-vaccination reactogenic symptoms - the physical manifestation of the inflammatory response to vaccination^3^—is often a contributing factor to such hesitancy^4^. Investigating the frequency with which post-vaccination reactogenic symptoms are experienced, risk factors for experiencing them and associations with vaccine immunogenicity has potential to yield information that will allow potential vaccinees to assess their risk of experiencing such symptoms, and to understand the significance of their presence or absence.

Existing studies investigating reactogenicity to first and second doses of SARS-CoV-2 vaccination have reported that post-vaccination symptoms are more commonly reported by women vs. men, in younger vs. older people, and in those who have had previous SARS-CoV-2 infection vs. those who have not^5–9^. However, the range of potential determinants explored by these studies has been limited: none have explored the potential impact of pre-vaccination anxiety or depression, specific comorbidities, medications or socio-economic factors which may either directly influence risk of reactogenic symptoms, or confound associations with age and sex. Additionally, there is controversy regarding the relationship between post-vaccination symptoms from a full course of vaccination and SARS-CoV-2 vaccine immunogenicity, with some studies showing no relationship between post-vaccination symptoms and titres of antibodies to the SARS-CoV-2 spike (S) glycoprotein^7,10^, one reporting a positive association overall^8^, and another reporting a positive association in men, but not in women^11^. These studies were relatively small and were not population based, limiting both power and generalisability of their findings.

To address these limitations, we conducted a large, population-based study in United Kingdom (UK) adults receiving SARS-CoV-2 vaccines, capturing detailed information on multiple potential determinants of reactogenic symptoms, and assaying combined IgG, IgA and IgM responses to SARS-CoV-2 spike protein before and after vaccination.

## Results

### Participant flow and characteristics

Of the 17,796 participants who completed the COVIDENCE UK baseline questionnaire by September 2021, we excluded those who had no pre-vaccination serology (*n* = 6343) or who had not completed a full vaccination regimen (*n* = 2273). We also excluded those with missing data for independent variables required for minimal adjustment in the regression models (*n* = 177), resulting in 9003 participants being eligible for this analysis (Supplementary Fig. 1, Supplementary Material). Selected baseline characteristics of participants included in the analysis are presented in Table 1. The mean age of those contributing data to analyses was 61.3 years (range: 16.6–88.0), 70.7% were female, 96.4% identified their ethnic origin as White, and 88.4% were living in England. A total of 6322 (70.2%) participants experienced at least one post-vaccination symptom. The mean number of days from the date of second vaccine dose to date of dried blood spot was 58.2 (s.d. 22.4). This differed slightly for participants who did vs. did not report systemic symptoms after vaccination (56.2 vs. 61.2 days, *P* < 0.001), but was no different for those who did vs. did not report local symptoms after vaccination (57.9 vs. 58.6 days, *P* = 0.23).

**Table 1:** Participant characteristics at baseline (*n* = 9003).

	Characteristic	*N* (%)
Age, years	16–29.99	120 (1.3)
	30–39.99	341 (3.8)
	40–49.99	800 (8.9)
	50–59.99	2192 (24.4)
	60–69.99	3617 (40.2)
	≥70.00	1933 (21.5)
Sex	Female	6367 (70.7)
	Male	2363 (29.3)
Ethnicity	White	8676 (96.4)
	Mixed/multiple/other ethnic groups	197 (2.2)
	Asian/Asian British	94 (1.0)
	Black/African/Caribbean/Black British	36 (0.4)
Country of residence	England	7961 (88.4)
	Northern Ireland	139 (1.5)
	Scotland	563 (6.3)
	Wales	340 (3.8)
Highest educational level attained	Primary/Secondary	978 (10.9)
	Higher/further (A levels)	1265 (14.1)
	College	3991 (44.4)
	Post-graduate	2762 (30.7)
Body mass index^a^	<25, kg/m^2^	4369 (48.6)
	25–30, kg/m^2^	2907 (32.4)
	>30, kg/m^2^	1709 (19.0)
Self-reported general health	Excellent	1878 (20.9)
	Very good	3590 (39.88)
	Good	2326 (25.8)
	Fair	949 (10.5)
	Poor	260 (2.9)
Tobacco smoking status	Not a current smoker	8633 (95.9)
	Current smoker	370 (4.1)
E-cigarette status^b^	Not a current vaper	8778 (97.7)
	Current smoker	207 (2.3))
Alcohol consumption	None	2349 (26.1)
	1–7 units	3189 (35.4)
	8–14 units	1853 (20.6)
	15–21 units	901 (10.0)
	22–28 units	403 (4.5)
	>28 units	308 (3.4)
Vaccine type	2 doses ChAdOx1	5988 (66.5)
	2 doses BNT162b2	2864 (31.8)
	2 doses MRNA-1273	59 (0.66)
	2 doses Other^c^	92 (1.02)
Month of dose for first dose	Q1 (Jan–Mar)	8262 (91.8)
	Q2 (Apr–Jun)	625 (6.9)
	Q3 (Jul–Sep)	1 (0.01)
	Q4 (Oct–Dec)	115 (1.3)
Month of dose for second dose	Q1 (Jan–Mar)	751 (8.3)
	Q2 (Apr–Jun)	7975 (88.6)
	Q3 (Jul–Sep)	274 (3.0)
	Q4 (Oct–Dec)	3 (0.03)

^a^Body mass index missing for 18 participants.

^b^Vape status missing for 18 participants.

^c^Single dose Janssen (*n* = 12), 2 doses Novavax (*n* = 6), 2 doses Valneva (*n* = 3), Comcov2 trial (*n* = 1), Not sure/do not know.

### Incidence of reactive symptoms by dose order and vaccine type

**Fig. 1:**
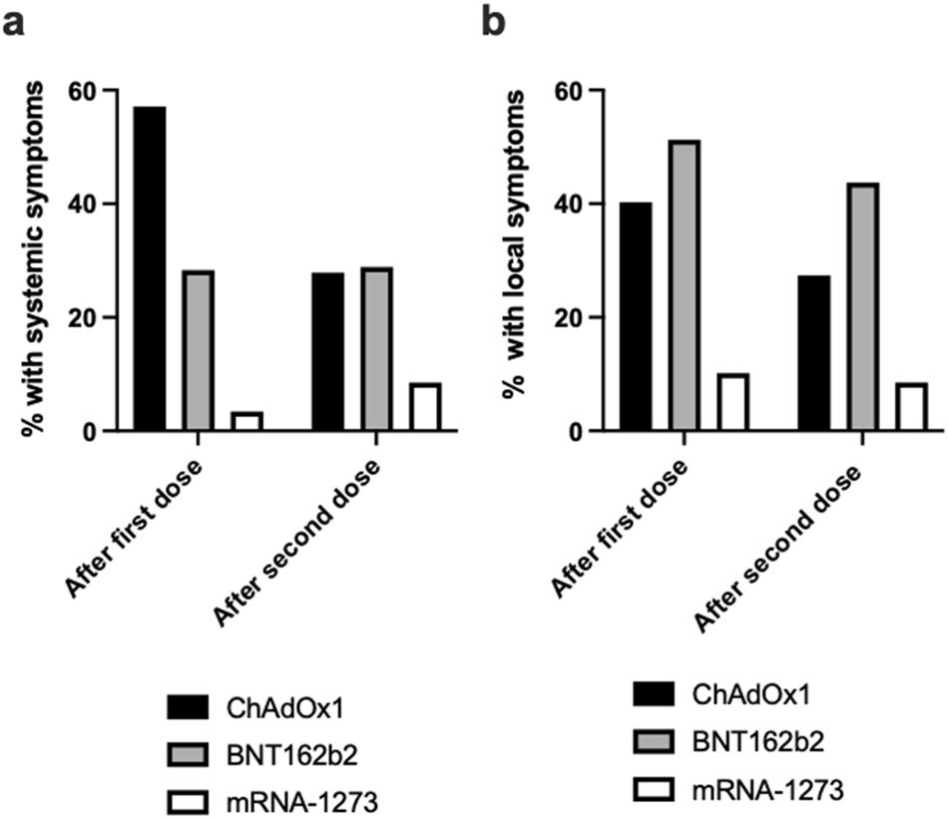
Proportions of participants experiencing systemic or local reactive symptoms after receiving first and second doses of ChAdOx1, BNT162b2, and mRNA-1273 vaccines. Panel **a** shows systemic symptoms are more common with ChAdOx1 vaccine following first dose. Panel **b** shows local symptoms are more common with BNT162b2 vaccine following second dose.

Figure 1 presents the proportions of participants reporting systemic and local symptoms by dose order and vaccine type. Systemic symptoms following first dose were most commonly reported by participants who received ChAdOx1 (57.1%), followed by BNT162b2 (28.3%) and mRNA-1273 (3.4%; *P* < 0.001). Following the second dose, the proportion of participants experiencing systemic symptoms was similar for ChAdOx1 (27.9%) vs BNT162b2 (28.9%), but lower for mRNA-1273 (8.5%; *P* = 0.002). Local symptoms following first dose were most common for BNT162b2 (51.3%), followed by ChAdOx1 (40.3%) and mRNA-1273 (10.2%; *P* < 0.001). Local symptoms after the second dose were reported less frequently than after the first dose, with a similar distribution according to vaccine type as seen with the first dose (BNT162b2 43.7% vs. ChAdOx1 27.4% vs. mRNA-1273 8.5%; *P* < 0.001).

### Determinants of systemic reactive symptoms following first vaccine dose

After adjustment for age and sex only, 23 variables were found to associate with risk of experiencing systemic symptoms following a first vaccine dose with *P* < 0.10 (Table 2), and 14 did not (Supplementary Table 3, Supplementary Material). After inclusion of the former group of factors in a multivariable model, the following variables were independently associated with higher risk of reporting systemic symptoms after first vaccine dose: time of year (aOR 3.79, 95% CI: 3.01–4.76, for Q1 vs. Q2; aOR 4.28, 95% CI: 2.69–6.80, for Q4 vs. Q2), poorer self-rated general health (aOR per category of worsening health 1.15, 95% CI: 1.10–1.21), pre-vaccination SARS-CoV-2 seropositivity with symptoms vs. pre-vaccination SARS-CoV-2 seronegativity (aOR 2.23, 95% CI: 1.78–2.81), presence vs. absence of self-rated anxiety or depression (aOR 1.24, 95% CI: 1.12–1.39), presence vs. absence of atopic disease (aOR 1.20, 95% CI: 1.08–1.34), presence vs. absence of arterial disease (aOR 1.31, 95% CI: 1.06–1.63), presence vs. absence of kidney disease (aOR 1.40, 95% CI: 1.01–1.93), and use vs. no use of multivitamin supplements (aOR 1.41, 95% CI: 1.26–1.58). Lower risk of developing systemic post-vaccination symptoms following first dose was independently associated with administration of mRNA vaccines vs. ChAdOx1 (aOR 0.29, 95% CI: 0.26–0.32 for BNT162b2 vs. ChAdOx1; aOR 0.06, 95% CI: 0.01–0.26 for mRNA-1273 vs. ChAdOx1), greater age (aOR per additional 10 years of age 0.85, 95% CI: 0.81–0.90), male vs. female sex at birth (aOR 0.59, 95% CI: 0.53–0.65), presence vs. absence of previous cancer (aOR 0.83, 95% CI: 0.71–0.97), use vs. no use of statins (aOR 0.84, 95% CI: 0.74–0.97), and use vs. no use of ACE inhibitors (aOR 0.80, 95% CI: 0.69–0.94).

**Table 2:** Incidence and determinants of systemic reactive symptoms after a first dose of SARS-CoV-2 vaccine.

			Minimally adjusted		Fully adjusted	
		*N* (%) symptomatic	aOR (95% CI)	*P*	aOR (95% CI)	*P*
Vaccine type	ChAdOx1	3420/5988 (57.0)	1.00		1.00	
	BNT162b2	810/2864 (28.3)	0.31 (0.28–0.34)	<0.001	0.29 (0.26–0.32)	<0.001
	MRNA-1273	2/59 (3.4)	0.03 (0.01–0.11)	<0.001	0.06 (0.01–0.26)	<0.001
	Other	30/92 (43.6)	0.34 (0.22–0.54)	<0.001	0.39 (0.4–0.62)	<0.001
Month of dose	Q1 (Jan–Mar)	4080/8262 (49.4)	4.14 (3.33–5.14)	<0.001	3.79 (3.01–4.76)	<0.001
	Q2 (Apr–Jun)	144/625 (23.0)	1.00		1.00	
	Q3 (Jul–Sep)	0/1 (0.00)	N/A		N/A^a^	
	Q4 (Oct–Dec)	38/1125 (31.3)	1.94 (1.24–3.02)	0.003	4.28 (2.69–6.80)	<0.001
Age, years	16–29.99	45/120 (37.5)	1.00		1.00	
	30–39.99	121/341 (35.5)	0.92 (0.59–1.42)	0.69	0.82 (0.49–1.33)	0.44
	40–49.99	373/800 (46.6)	1.42 (0.96–2.12)	0.08	0.90 (0.57–1.42)	0.65
	50–59.99	1286/2192 (58.7)	2.36 (1.61–3.45)	<0.001	1.03 (0.66–1.61)	0.90
	60–69.99	1716/3617 (47.4)	1.56 (1.07–2.28)	0.021	0.72 (0.46–1.12)	0.14
	≥70.00	721/1933 (37.3)	1.10 (0.75–1.61)	0.64	0.58 (0.37–0.92)	0.020
	*P* for trend				0.85 (0.81–0.90)	<0.001
Sex	Female	3297/6367 (51.8)	1.00		–	–
	Male	965/2636 (36.6)	0.57 (0.52–0.63)	<0.001	0.59 (0.53–0.65)	<0.001
IMD rank, quartile	Q1 (most deprived)	906/2030 (44.7)	0.84 (0.75–0.95)	0.006	0.90 (0.79–1.02)	0.10
	Q2	1020/2160 (47.2)	0.95 (0.84–1.07)	0.39	0.95 (0.84–1.08)	0.44
	Q3	1129/2343 (48.2)	1.00 (0.89–1.12)	0.96	1.00 (0.89–1.13)	0.97
	Q4 (least deprived)	1182/2430 (48.7)	1.00		–	–
	P for trend				0.96 (0.92–1.00)	0.070
Tobacco smoking status	Not current smoker	4097/8633 (47.5)	1.00		1.00	
	Current smoker	165/370 (44.7)	0.83 (0.67–1.03)	0.09	0.80 (0.64–1.01)	0.06
Alcohol, units/wk	0	1170/2349 (49.8)	1.00		1.00	
	1–7	1530/3189 (48.0)	0.95 (0.85–1.05)	0.31	1.01 (0.90–1.13)	0.91
	8–14	861/1853 (46.5)	0.93 (0.82–1.05)	0.25	1.02 (0.90–1.18)	0.68
	15–21	398/901 (44.2)	0.86 (0.73–1.01)	0.06	0.91 (0.77–1.08)	0.30
	22–28	178/403 (44.3)	0.91 (0.73–1.14)	0.42	0.98 (0.78–1.24)	0.89
	>28	125/308 (40.6)	0.80 (0.62–1.03)	0.08	0.84 (0.65–1.10)	0.21
	*P* for trend				0.98 (0.94–1.02)	0.26
Self-rated general health	Excellent	801/18,758 (42.7)	1.00		1.00	
	Very good	1620/3590 (45.1)	1.15 (1.02–1.29)	0.019	1.13 (1.00–1.28)	0.05
	Good	1162/2326 (50.0)	1.37 (1.21–1.56)	<0.001	1.38 (1.20–1.58)	<0.001
	Fair	530/949 (55.9)	1.67 (1.42–1.96)	<0.001	1.53 (1.28–1.83)	<0.001
	Poor	149/260 (57.3)	1.74 (1.33–2.27)	<0.001	1.54 (1.14–2.07)	0.005
	*P* for trend				1.15 (1.10–1.21)	<0.001
Pre-vaccination SARS-CoV-2 status	Seronegative	3560/7640 (46.6)	1.00		1.00	
	Seropositive asymptomatic	439/958 (45.8)	0.99 (0.86–1.13)	0.83	0.94 (0.81–1.09)	0.42
	Seropositive symptomatic	263/405 (64.9)	2.01 (1.62–2.48)	<0.001	2.23 (1.78–2.81)	<0.001
	*P* for trend				1.26 (1.15–1.38)	<0.001
Self-rated anxiety or depression	No	3040/6744 (45.1)	1.00		1.00	
	Yes	1220/2253 (54.2)	1.35 (1.23–1.49)	<0.001	1.24 (1.12–1.39)	<0.001
Asthma	No	3489/7552 (46.2)	1.00		1.00	
	Yes	773/1451 (53.3)	1.29 (1.15–1.45)	<0.001	1.12 (0.95–1.32)	0.18
Atopic disease^b^	No	3041/6684 (45.5)	1.00		1.00	
	Yes	1221/2319 (52.7)	1.28 (1.16–1.41)	<0.001	1.20 (1.08–1.34)	0.001
Arterial disease	No	4040/8514 (47.5)	1.00		1.00	
	Yes	222/489 (45.4)	1.21 (1.00–1.47)	0.049	1.31 (1.06–1.63)	0.014
Kidney disease	No	4164/8821 (47.2)	1.00		1.00	
	Yes	98/182 (53.9)	1.47 (1.09–1.99)	0.012	1.40 (1.01–1.93)	0.043
Cancer	Never	3891/8117 (47.9)	1.00		1.00	
	Previous	346/809 (42.8)	0.84 (0.72–0.97)	0.021	0.83 (0.71–0.97)	0.019
	Active	25/77 (32.5)	0.64 (0.39–1.05)	0.078	0.62 (0.37-1.05)	0.076
Statins	No	3620/7,377 (49.1)	1.00		1.00	
	Yes	642/1626 (39.5)	0.87 (0.77–0.98)	0.022	0.84 (0.74–0.97)	0.014
ACE inhibitors	No	3903/8101 (48.2)	1.00		1.00	
	Yes	359/902 (39.8)	0.82 (0.7–0.95)	0.008	0.80 (0.69–0.94)	0.007
Inhaled corticosteroids	No	3951/8415 (47.0)	1.00		1.00	
	Yes	311/588 (52.9)	1.24 (1.04–1.47)	0.015	0.98 (0.78–1.25)	0.89
Systemic immunosuppressants	No	4051/8607 (47.1)	1.00		1.00	
	Yes	211/396 (53.3)	1.29 (1.05–1.59)	0.014	1.10 (0.87–1.38)	0.423
Inhaled bronchodilators	No	3807/8160 (46.6)	1.00		1.00	
	Yes	453/837 (54.1)	1.31 (1.14–1.52)	<0.001	1.49 (0.63–3.50)	0.37
Beta-2 adrenergic agonists	No	3,822/8,192 (46.7)	1.00		1.00	
	Yes	440/811 (54.3)	1.31 (1.13–1.51)	<0.001	0.66 (0.27–1.60)	0.36
Multivitamin supplement	No	3258/7168 (45.5)	1.00		1.00	
	Yes	1004/1835 (54.7)	1.42 (1.28–1.58)	<0.001	1.41 (1.26–1.58)	<0.001
Vitamin D supplement	No	3213/6690 (48.1)	1.00		1.00	
	Yes	1049/2133 (45.3)	1.81 (0.97–3.38)	0.06	0.92 (0.83–1.02)	0.12

^a^ORs were not calculated where fewer than 5 participants experienced an event.

^b^Atopic disease defined by atopic eczema/dermatitis and/or hayfever/allergic rhinitis.

Given that we showed no association between asymptomatic pre-vaccination SARS-CoV-2 infection and risk of systemic symptoms after a first vaccine dose (aOR 0.94, 95% CI: 0.81–1.09, Table 2), we conducted an exploratory analysis to compare pre-vaccination anti-S titres between participants who experienced symptomatic vs. asymptomatic SARS-CoV-2 infection prior to vaccination: the former group had higher anti-S titres than the latter (*P* < 0.001, Supplementary Fig. 2).

In another exploratory analysis, we investigated whether these factors also associated independently with the mean total number of different systemic symptoms reported by the subset of participants reporting at least one such symptom. Results are displayed in Supplementary Table 4, Supplementary Material. Higher mean number of systemic symptoms reported associated independently with younger age (*P* for trend 0.01), female vs. male sex (1.62 vs. 1.54, *P* = 0.028), symptomatic SARS-CoV-2 infection vs. no SARS-CoV-2 infection pre-vaccination (1.76 vs. 1.58, *P* = 0.013), administration of ChAdOx1 vs. BNT162b2 (1.69 vs. 1.20, *P* < 0.001) and presence vs. absence of atopic disease (1.66 vs. 1.58, *P* = 0.019).

Determinants of systemic symptoms following a second dose of vaccine were broadly similar, in that administration of BNT162b2 vs. ChAdOx1, vaccination in Q1 vs. Q2, poorer vs. better self-rated general health, pre-vaccination SARS-CoV-2 seropositivity with symptoms vs. pre-vaccination SARS-CoV-2 seronegativity, presence vs. absence of self-rated anxiety or depression, presence vs. absence of atopic disease, and use vs. no use of multivitamin supplements associated with higher risk of reporting symptoms and greater age, and male vs. female sex at birth all associated with lower risk. Additionally, a shorter inter-dose interval associated with reduced risk of systemic symptoms after the second vaccine dose (aOR 0.61, 95% CI: 0.46–0.83 for <6 weeks vs. >10 weeks; aOR 0.86, 95% CI: 0.76–0.96 for 6–10 weeks vs. >10 weeks), as did higher alcohol intake (aOR per increasing category 0.93, 95% CI: 0.89–0.96) and use vs. no use of anticholinergic medication (aOR 0.73, 95% CI: 0.53–0.99; Supplementary Table 5, Supplementary Material).

### Determinants of local reactive symptoms following first vaccine dose

After adjustment for age and sex only, 33 variables were found to associate with risk of experiencing local symptoms following a first vaccine dose with *P* < 0.10 (Table 3) and 8 were not (Supplementary Table 6, Supplementary Material). After inclusion of all of these factors in a multivariable model, the following variables were independently associated with higher risk of reporting local symptoms after first dose: administration of BNT162b2 vs. ChAdOx1 (aOR 1.95 95% CI: 1.74–2.18), administration of vaccine at lunchtime (i.e. 12:00–14:00) vs. morning (i.e. before 12:00; aOR 1.23, 95% CI: 1.07–1.41), vegetarian vs. unrestricted diet (aOR 1.36, 95% CI: 1.05–1.76), poorer vs. better self-rated general health (aOR per worsening health category 1.14, 95% CI: 1.08–1.21), pre-vaccination SARS-CoV-2 seropositivity with symptoms vs. pre-vaccination SARS-CoV-2 seronegativity (aOR 1.84, 95% CI: 1.43–2.38), presence vs. absence of self-rated anxiety or depression (aOR 1.17, 95% CI: 1.03–1.32), presence vs. absence of atopic disease (aOR 1.15, 95% CI: 1.02–1.30), BCG vaccinated vs. not (aOR 1.21, 95% CI: 1.04–1.41), and use vs. no use of multivitamin supplements (aOR 1.41, 95% CI: 1.26–1.58). Lower risk of developing local post-vaccination symptoms following first dose was independently associated with greater age (aOR per additional 10 years of age 0.62, 95% CI: 0.59–0.66), male vs. female sex at birth (aOR 0.48, 95% CI: 0.43–0.54), and lower educational level (aOR per decreasing education category 0.94, 95% CI: 0.89–0.99). Determinants of local symptoms following a second dose of vaccine were broadly similar, in that administration of BNT162b2 vs. ChAdOx1, poorer vs. better self-rated general health, pre-vaccination SARS-CoV-2 seropositivity with symptoms vs. pre-vaccination SARS-CoV-2 seronegativity, presence vs. absence of self-rated anxiety or depression, presence vs. absence of atopic disease, and use vs. no use of multivitamin supplements associated with higher risk of reporting local symptoms and greater age, male vs. female sex at birth and lower educational level associated with lower risk. Additionally, use vs. no use of H2-receptor antagonists associated with increased risk of local symptoms after second dose (aOR 2.05, 95% CI: 1.21–3.47), while use vs. no use of anticholinergics (aOR 0.73, 95% CI: 0.53–0.99) and previous history of cancer vs. no such history (aOR 0.83, 95% CI: 0.70–0.99) both associated with reduced risk; Supplementary Table 7, Supplementary Material).

**Table 3:** Incidence and determinants of local reactive symptoms after a first dose of SARS-CoV-2 vaccine.

			Minimally adjusted		Fully adjusted	
		*N* (%) symptomatic	aOR (95% CI)	*P*	aOR (95% CI)	*P*
Vaccine type	ChAdOx1	2414/5988 (40.3)	1.00		1.00	
	BNT162b2	1470/2864 (51.3)	1.78 (1.62–1.96)	<0.001	1.95 (1.74–2.18)	<0.001
	MRNA-1273	6/59 (10.2)	0.16 (0.07–0.38)	<0.001	N/A	N/A
	Other	24/92 (26.1)	0.50 (0.31–0.81)	0.005	0.47 (0.27–0.82)	0.008
Timing	Before 12:00	1520/3193 (47.6)	1.00			1.00
	12:00–14:00	745/1425 (52.3)	1.23 (1.08–1.41)	0.002	1.23 (1.07–1.41)	0.004
	14:00–17:00	1097/2200 (49.9)	1.09 (0.98–1.22)	0.123	1.05 (0.93–1.18)	0.45
	After 17:00	509/1030 (49.4)	1.03 (0.89–1.19)	0.720	1.00 (0.85–1.17)	0.99
Month of dose	Q1 (Jan–Mar)	3722/8262 (45.1)	5.07 (4.04–6.35)	<0.001	1.11 (0.81–1.52)	0.51
	Q2 (Apr–Jun)	129/625 (20.6)	1.00		1.00	
	Q3 (Jul–Sep)	0/1 (0.00)	N/A		N/A	
	Q4 (Oct–Dec)	63/115 (54.8)	6.90 (4.46–10.7)	<0.001	0.89 (0.52–1.53)	0.68
Age, years	16–29.99	50/120 (41.7)	1.00		1.00	
	30–39.99	128/341 (37.5)	0.84 (0.55–1.29)	0.43	0.51 (0.21–1.28)	0.15
	40–49.99	389/800 (48.6)	1.29 (0.87–1.91)	0.20	0.46 (0.20–1.09)	0.08
	50–59.99	1164/2192 (53.1)	1.57 (1.08–2.29)	0.019	0.26 (0.11–0.60)	0.002
	60–69.99	1546/3617 (42.7)	1.09 (0.75–1.58)	0.66	0.17 (0.07–0.38)	<0.001
	≥70.00	637/1933 (33.0)	0.77 (0.53-1.13)	0.18	0.10 (0.04–0.23)	<0.001
	*P* for trend				0.62 (0.59–0.66)	<0.001
Sex	Female	3104/6367 (48.8)	1.00		1.00	
	Male	810/2636 (30.7)	0.50 (0.46–0.55)	<0.001	0.48 (0.43–0.54)	<0.001
Education	Primary/Secondary	381/978 (39.0)	0.82 (0.71–0.96)	0.012	0.78 (0.66–0.94)	0.009
	Higher/Further	553/1265 (43.7)	0.95 (0.83–1.09)	0.48	0.97 (0.82–1.34)	0.68
	College	1728/3991 (43.3)	0.94 (0.85–1.04)	0.24	0.96 (0.85–1.08)	0.50
	Post-graduate	1248/2762 (45.2)	1.00		1.00 (ref)	
	P for trend				0.94 (0.89–0.99)	0.024
Vigorous physical exercise, hr/wk	0	1529/3359 (45.5)	1.19 (1.06–1.32)	0.002	1.02 (0.89–1.17)	0.74
	1–3	1450/3323 (43.6)	1.09 (0.97–1.21)	0.15	1.05 (0.92–1.19)	0.49
	≥4	926/2301 (40.3)	1.00		1.00	
	*P* for trend				0.99 (0.92–1.06)	0.78
Light physical exercise, hr/wk	0–4	1305/2832 (46.1)	1.16 (1.04–1.29)	0.006	1.05 (0.92–1.19)	0.49
	5–9	1311/2983 (44.0)	1.07 (0.96–1.18)	0.24	1.07 (0.95–1.21)	0.27
	≥10	1293/3172 (40.8)	1.00		1.00	
	*P* for trend				0.98 (0.91–1.04)	0.46
Dietary restrictions	None	3679/8500 (43.3)	1.00			1.00
	Vegetarian	196/397 (49.4)	1.16 (0.95–1.43)	0.15	1.36 (1.05–1.76)	0.019
	Vegan	3679/8500 (43.3)	0.70 (0.47–1.04)	0.080	0.64 (0.40–1.03)	0.067
Self-rated general health	Excellent	711/1878 (37.8)	1.00		1.00	
	Very good	1491/3590 (41.5)	1.21 (1.08–1.36)	0.001	1.19 (1.04–1.36)	0.013
	Good	1099/2326 (47.3)	1.50 (1.32–1.70)	<0.001	1.43 (1.23–1.68)	<0.001
	Fair	481/949 (50.7)	1.64 (1.40–1.93)	<0.001	1.49 (1.21–1.83)	<0.001
	Poor	132/260 (50.8)	1.60 (1.23–2.09)	0.001	1.41(1.00–1.99)	0.048
	*P* for trend				1.14 (1.08–1.21)	<0.001
Body mass index, kg/m^2^	<25	1882/4369 (43.1)	1.00		1.00	
	25–30	1226/2907 (42.2)	1.04 (0.94–1.14)	0.38	0.95 (0.85–1.07)	0.38
	>30	799/1709 (46.8)	1.12 (1.00–1.27)	0.05	0.88 (0.76–1.02)	0.08
Pre-vaccination SARS-CoV-2 status	Seronegative	3270/7640 (42.8)	1.00			1.00
	Seropositive asymptomatic	407/958 (42.5)	1.00 (0.87–1.15)	0.96	0.96 (0.81–1.12)	0.58
	Seropositive symptomatic	237/405 (58.5)	1.74 (1.41–2.14)	<0.001	1.84 (1.43–2.38)	<0.001
	*P* for trend				1.19 (1.08–1.32)	0.001
Self-rated anxiety or depression	No	2806/6744 (41.6)	1.00		1.00	
	Yes	1106/2253 (49.1)	1.23 (1.11–1.35)	<0.001	1.17 (1.03–1.32)	0.018
Asthma	No	3206/7552 (42.5)	1.00		1.00	
	Yes	708/1451 (48.8)	1.23 (1.10–1.38)	<0.001	1.17 (0.97–1.41)	0.10
Atopic disease^a^	No	2781/6684 (41.6)	1.00		1.00	
	Yes	1133/2319 (48.8)	1.27 (1.15–1.40)	<0.001	1.15 (1.02–1.30)	0.019
Diabetic status	No diabetes	3612/8278 (43.6)	1.00		1.00	
	Pre-diabetes	120/291 (41.0)	1.03 (0.81–1.32)	0.79	0.88 (0.67–1.17)	0.38
	Type 1 diabetes	35/61 (57.4)	1.84 (1.09–3.09)	0.022	1.02 (0.57–1.84)	0.94
	Type 2 diabetes	141/363 (38.6)	0.99 (0.79–1.24)	0.95	0.94 (0.71–1.25)	0.68
Heart disease	No	3769/8642 (43.6)	1.00		1.00	
	Yes	145/361 (40.2)	1.23 (0.99–1.54)	0.07	0.78 (0.49–1.22)	0.27
Arterial disease	No	3708/8514 (43.5)	1.00		1.00	
	Yes	206/489 (42.1)	1.33 (1.10–1.62)	0.004	1.43 (0.90–2.28)	0.13
Major neurological condition	No	3802/8752 (43.4)	1.00		1.00	
	Yes	112/251 (44.6)	1.29 (1.00–1.68)	0.05	1.00 (0.66–1.51)	1.00
Immunodeficiency	No	3883/8950 (43.4)	1.00		1.00	
	Yes	31/53 (58.5)	2.03 (1.16–3.55)	0.013	1.80 (0.92-3.47)	0.09
Statins	No	3272/7377 (44.4)	1.00		1.00	
	Yes	642/1626 (39.5)	1.18 (1.04–1.33)	0.008	1.09 (0.94–1.28)	0.26
Proton pump inhibitors	No	3354/7752 (43.3)	1.00		1.00	
	Yes	560/1251 (44.8)	1.20 (1.06–1.35)	0.005	0.98 (0.84–1.14)	0.77
Inhaled corticosteroids	No	3622/8415 (43.0)	1.00		1.00	
	Yes	292/588 (49.7)	1.27 (1.07–1.51)	0.006	1.02 (0.79–1.32)	0.89
SSRIs	No	3619/8430 (42.9)	1.00		1.00	
	Yes	295/573 (51.5)	1.22 (1.03–1.46)	0.022	1.06 (0.86–1.32)	0.57
Thiazides	No	3782/8708 (43.4)	1.00		1.00	
	Yes	132/295 (44.8)	1.25 (0.98–1.59)	0.07	1.24 (0.94–1.63)	0.14
Inhaled bronchodilators	No	3807/8160 (46.6)	1.00		1.00	
	Yes	453/837 (54.1)	1.31 (1.14–1.52)	<0.001	1.49 (0.63–3.50)	0.37
Sodium-glucose co-transporter-2 (SGLT2) inhibitors	No	3901/8955 (43.6)	1.00		1.00	
	Yes	13/48 (27.1)	0.57 (0.30–1.09)	0.09	0.48 (0.22–1.05)	0.07
Anti-platelet drugs	No	3666/8404 (43.6)	1.00		1.00	
	Yes	248/599 (41.4)	1.26 (1.06–1.50)	0.011	0.88 (0.54–1.44)	0.61
Beta-2 adrenergic agonists	No	3822/8192 (46.7)	1.00		1.00	
	Yes	440/811 (54.3)	1.31 (1.13–1.51)	<0.001	0.66 (0.27–1.60)	0.36
Aspirin	No	3716/8531 (43.6)	1.00		1.00	
	Yes	198/472 (42.0)	1.29 (1.06-1.56)	0.012	1.22 (0.74–2.02)	0.44
BCG vaccinated	No	414/1087 (38.1)	1.00		1.00	
	Yes	3197/7084 (45.1)	1.25 (1.09–1.43)	0.001	1.21 (1.04–1.41)	0.013
Multivitamin supplement	No	3258/7168 (45.5)	1.00		1.00	
	Yes	1004/1835 (54.7)	1.42 (1.28–1.58)	<0.001	1.41 (1.26–1.58)	<0.001
Vitamin D supplement	No	3213/6690 (48.1)	1.00		1.00	
	Yes	1049/2133 (45.3)	1.81 (0.97–3.38)	0.06	0.92 (0.83–1.02)	0.12

^a^Atopic disease defined by atopic eczema/dermatitis and/or hayfever/allergic rhinitis.

### Association between reactive symptoms and post-vaccination anti-S titres

Table 4 displays mean post-vaccination antibody titres by frequency of post-vaccination symptoms, stratified by vaccine type (ChAdOx1 vs BNT162b2) and adjusted for duration between date of second dose and date of post-vaccination antibody measurement and pre-vaccination anti-S titres. For both ChAdOx1 and BNT162b2, a dose–response relationship between increasing symptom frequency and higher post-vaccination antibody titres was observed for both local and systemic symptoms (*P* for trend ≤ 0.02). A sensitivity analysis excluding participants who had SARS-CoV-2 infection prior to vaccination (as evidenced by presence of anti-S antibodies and/or a positive RT-PCR or antigen test for SARS-CoV-2) yielded consistent findings (Supplementary Table 8, Supplementary Material).

**Table 4:** Post-vaccination antibody titres by vaccine type and post-vaccination reactive symptoms.

	*N*	Mean titre (s.d.)	Adjusted mean difference (95% CI)^a^	*P*	*P* for trend
Local reactive symptoms					
Never	2150	2.64 (2.23)	– (ref)	–	0.007^b^
After one dose only	1212	2.78 (2.22)	0.05 (−0.10 to 0.19)	0.534	
After both doses	995	3.03 (2.76)	0.23 (0.07 to 0.38)	0.004	
Systemic reactive symptoms					
Never	1437	2.54 (2.06)	– (ref)	–	<0.001^c^
After one dose only	1812	2.83 (2.42)	0.25 (0.11 to 0.39)	0.001	
After both doses	1108	2.95 (2.25)	0.33 (0.17 to 0.49)	<0.001	
Local reactive symptoms					
Never	720	4.54 (3.30)	– (ref)	–	0.029^d^
After one dose only	501	4.78 (3.06)	0.30 (−0.05 to 0.64)	0.097	
After both doses	805	4.93 (3.21)	0.34 (0.04 to 0.65)	0.027	
Systemic reactive symptoms					
Never	1120	4.49 (3.00)	– (ref)	–	<0.001^e^
After one dose only	548	4.81 (3.13)	0.30 (−0.01 to 0.61)	0.058	
After both doses	358	5.51 (3.80)	0.75 (0.38 to 1.11)	<0.001	

^a^Adjusted for pre-vaccination anti-S titre and number of days from date of second vaccine dose to date of post-vaccination antibody measurement.

^b^Associated beta coefficient per increasing category 0.11 (95% CI: 0.03–0.18).

^c^Associated beta coefficient per increasing category 0.17 (95% CI: 0.09–0.25).

^d^Associated beta coefficient per increasing category 0.17 (95% CI: 0.02–0.32).

^e^Associated beta coefficient per increasing category 0.36 (95% CI: 0.19–0.53).

### Association between long COVID and risk of reactive symptoms following vaccination

Supplementary Table 9 presents results of an exploratory analysis restricted to participants with evidence of SARS-CoV-2 infection prior to vaccination, conducted to determine whether presence or absence of self-reported ‘long COVID’ associated with risk of reactive symptoms following SARS-CoV-2 vaccination. After adjustment for all covariates included in the final models described above, we found that self-reported long COVID associated independently with increased risk of both systemic and local reactive symptoms after a first vaccine dose (systemic, aOR 1.73, 95% CI: 1.12–3.35; local, aOR 1.94, 95% CI: 1.41–2.66). However, self-reported long COVID did not associate independently with risk of either systemic or local reactive symptoms after a second vaccine dose.

## Discussion

This large, prospective, population-based study represents the most comprehensive investigation of incidence, determinants and serological correlates of reactogenic symptoms following SARS-CoV-2 vaccination that has been conducted to date. We found that administration of ChAdOx1, younger age, female sex, poorer self-rated general health, pre-vaccination COVID-19, presence of self-rated anxiety or depression at enrolment, presence of atopic disease, and use of multivitamin supplements associated independently with increased odds of reporting both systemic and local symptoms following SARS-CoV-2 vaccination. A sub-set of these factors (younger age, female sex, pre-vaccination COVID-19, administration of ChAdOx1 and presence of atopic disease) also associated independently with a higher mean number of systemic symptoms reported. Those who received BNT162b2 vaccine, as compared to those who received ChAdOx1, were more likely to report local reactogenic symptoms and less likely to report systemic symptoms. Importantly, for both ChAdOx1 and BNT162b2, we found a dose-response relationship between increasing frequency of both local and systemic reactogenic symptoms and higher post-vaccination anti-S titres, that persisted when we excluded participants who had evidence of SARS-CoV-2 infection prior to vaccination.

Our findings are in keeping with those of other studies that have reported increased risk of post-vaccination symptoms in women vs. men^6,7,11^, in younger vs. older people^5–8^ and in those who had COVID-19 prior to vaccination^6^. However, we advance knowledge in this area by showing that participants who had asymptomatic SARS-CoV-2 infection prior to vaccination (as evidenced by presence of anti-S antibodies in the absence of any self-report of COVID-19 symptoms) were not at higher risk of post-vaccination symptoms than those who were anti-S seronegative prior to vaccination. This might reflect the presence of weaker pre-vaccination memory responses to SARS-CoV-2 in those who had asymptomatic vs. symptomatic SARS-CoV-2 infection, as evidenced by our demonstration of lower pre-vaccination anti-S titres in the former group. We also show for the first time that those reporting anxiety or depression at enrolment, those taking multivitamin supplements and vegetarians are more likely to report post-vaccination symptoms. The former association may reflect increased perception of physical symptoms that has previously been reported in people with anxiety and depression^12^. The latter might arise as a result of immunomodulatory effects of micronutrient supplements, which could augment reactogenic symptoms by boosting vaccine immunogenicity^13^; alternatively, participants taking micronutrient supplements or who maintain a vegetarian diet may be more health-conscious^14,15^, and thus have an increased awareness of post-vaccination symptoms. The incidence of systemic post-vaccination reactogenic symptoms reported in our study is broadly in keeping with that reported by other studies^5–8^, although incidence of local symptoms reported in this study is lower than for others^5–8^; this may reflect the relatively high proportion of participants receiving ChAdOx1 vs. mRNA vaccines in our study vs. others. Additionally, we highlight that our serological outcome was a combined IgG/IgA/IgM response to spike protein measured using a semiquantitative ELISA. This contrasts with other studies investigating associations between vaccine-related symptoms and serologic responses to SARS-CoV-2vaccination, which primarily use quantitative levels of IgG anti-S antibodies.

Our study has several strengths. Its prospective nature reduces the potential for reverse causation to explain the associations we report; for example, we show that anxiety is a prequel to reporting symptoms, and not a consequence of experiencing them. We investigated a comprehensive range of potential determinants of reactogenic symptoms, and captured detailed symptom profiles within days to weeks of vaccine administration, thereby reducing imprecision due to poor symptom recall. Additionally, the availability of pre- and post-vaccination anti-S status and COVID-19 symptom data allowed us to dissect apart the influence of symptomatic vs. asymptomatic SARS-CoV-2 infection on risk of reactogenic symptoms. A further strength is the use of an ELISA with high sensitivity and specificity that targets three different types of antibody (IgG/IgA/IgM), increasing the probability of identifying a past infection over other studies which primarily use quantitative levels of IgG anti-spike antibodies^16^. Additionally, we used dried blood spots for our sampling: although these yield a low volume of sample and their results may be impacted by variations in haematocrit, their use has been found to significantly reduce processing failures compared with microtubes^17^, which are currently used by large seroprevalence surveys^18^.

Our study also has some limitations. First, COVIDENCE UK is a self-selected cohort, and thus several groups—such as people younger than 30 years, people of lower socioeconomic status, and non-White ethnic groups—are under-represented. Second, in common with other studies in this field, we lack objective data relating to characterisation of reactions to vaccination; accordingly, the presence or absence of reported symptoms represents a function of the presence or absence of a physiological reaction to vaccination and the perception of that reaction. Third, those who had vaccines other than BNT162b2 or ChAdOx1 are under-represented, and this limited our power to investigate reactogenic symptoms following administration of other SARS-CoV-2 vaccines. Fourth, the duration of symptoms was not considered. Lastly, as with any observational study, we cannot exclude the possibility that the associations we report may be explained by residual and/or unmeasured confounding. We sought to minimise this by capture of, and mutual adjustment for, a comprehensive panel of potential confounders.

In conclusion, we found that factors that associate with lower risk of systemic post-vaccination symptoms include greater age, male sex, and receipt of an mRNA vaccine. Factors that associate with a higher risk of systemic post-vaccination symptoms include history of symptomatic SARS-CoV-2 infection prior to vaccination, presence of self-rated anxiety or depression and use of a multivitamin supplement. Finally, we found vaccine reactogenicity was associated with higher levels of antibody titres for both BNT162b2 and ChAdOx1 vaccines. Public communication of this message may provide a degree of reassurance to those who have experienced symptoms after SARS-CoV-2 vaccination and allow them to better understand the relationship between experiencing such symptoms and vaccine efficacy; this may in turn provide added motivation for them to receive further vaccine doses.

## Methods

### Study design, setting and participants

Our study was nested within COVIDENCE UK, which is a prospective longitudinal population-based observational study of coronavirus disease in the UK population (www.qmul.ac.uk/covidence)^16,17,19^. The study was launched on 1^st^ May 2020, and closed to enrolment on 6^th^ October 2021. Inclusion criteria for the cohort as a whole were age 16 years or more and residence in the UK at the time of enrolment. Participants were invited via a national media campaign to complete an online baseline questionnaire to capture information on potential symptoms of COVID-19 experienced since 1st February 2020, results of any COVID-19 tests, and details of a wide range factors that may influence risk of COVID-19 and response to vaccination, as described below. Follow-up questionnaires were administered at monthly intervals to capture incident test-confirmed COVID-19, potential symptoms of COVID-19, administration of SARS-CoV-2 vaccines and post-vaccination symptoms. Study participants were invited to provide dried blood spots for testing for combined IgG, IgA and IgM responses to the SARS-CoV-2 spike protein before and after SARS-CoV-2 vaccination as described elsewhere^16–18^. Analyses for the current study were restricted to the subset of participants whose pre-vaccination anti-S serostatus was determined; who completed a full primary course of SARS-CoV-2 vaccination; and who reported their post vaccination symptoms. This paper reports findings from analyses of data collected up to 1^st^ September 2021.

### Sponsorship, registration, ethics and reporting

The study was sponsored by Queen Mary University of London and approved by Leicester South Research Ethics Committee (ref 20/EM/0117). All participants provided informed consent by completing online consent forms. The study is registered with ClinicalTrials.gov (NCT04330599).

### Independent variables

Fifty-six putative risk factors for symptoms following administration of a SARS-CoV-2 vaccine were selected a priori, covering sociodemographic, occupational and lifestyle factors; longstanding medical conditions and prescribed medication use; Bacille Calmette Guérin vaccine status; and diet and supplemental micronutrient intake (Supplementary Tables 1 and 2Supplementary Material). These factors, which were obtained from the baseline and follow-up questionnaires, were included as independent variables in our models. To produce patient-level covariates for each class of medications investigated, participant responses were mapped to drug classes listed in the British National Formulary, or the DrugBank and Electronic Medicines Compendium databases if not explicitly listed in the British National Formulary, as previously described^19^. Index of Multiple Deprivation (IMD) 2019 scores were assigned according to participants’ postcodes, and categorised into quartiles. Duration of follow-up was defined as the number of days between the date of enrolment and the date of dried blood spot collection.

### Outcomes

The primary outcome for the current study was new symptoms following administration of the first dose of a SARS-CoV-2 vaccine. Symptoms were classified as being systemic (namely fatigue, headache, fever/high temperature [37.8 °C or greater] or myalgia) or local (namely tenderness, soreness, swelling, redness or a painful heavy feeling at the injection site and axillary or cervical lymphadenopathy). The questions used to capture these symptoms are presented in Supplementary Table 2, Supplementary Material. Secondary outcomes were incidence of symptoms after second vaccine doses, and titres of combined IgG, IgA and IgM anti-Spike antibody responses following vaccination, which were determined in eluates of postal dried blood spot samples as described below.

### Procedures for antibody testing

Procedures for the pre- and post-vaccination antibody studies are reported elsewhere^16,17^. Briefly, participants in these studies were sent kits containing instructions, lancets, and blood spot collection cards, to be posted back to the study team. Once returned, the samples were logged by the study team and sent in batches to the Clinical Immunology Service at the Institute of Immunology and Immunotherapy of the University of Birmingham (Birmingham, UK). Up to two more test kits were offered to participants whose initial samples were found to be insufficient for testing.

To elute antibody from DBS cards, we isolated individual pre-perforated DBS spots by using a sterile pipette tip and placed them into a universal tube at a ratio of 1 spot to 250 μL 0.05% phosphate-buffered saline (PBS)–Tween 20 (PBS-T) (PBS, xoid; Tween-20; Sigma-Aldrich, https://www.sigmaaldrich.com). We briefly vortexed and incubated tubes overnight at room temperature. We then harvested DBS eluate into a microtube and centrifuged it at 10,600×*g* for 10 min at room temperature. Eluates were stored at 4 °C for <14 days, prior to semi-quantitative determination of antibody titres. This was done using a commercially available ELISA that measures combined IgG, IgA, and IgM (IgGAM) responses to the SARS-CoV-2 trimeric spike glycoprotein (product code MK654; The Binding Site [TBS], Birmingham, UK). The SARS-CoV-2 spike used is a soluble, stabilised, trimeric glycoprotein truncated at the transmembrane region. 8,9 This assay has been CE-marked with 98.3% (95% CI: 96.4–99.4) specificity and 98.6% (92.6–100.0) sensitivity following RT-PCR-confirmed mild-to-moderate COVID-19 that did not result in hospitalisation.10 A cut-off ratio relative to the TBS cut-off calibrators was determined by plotting 624 pre-2019 negatives in a frequency histogram. A cut-off coefficient was then established for IgGAM (1.31), with ratio values classed as positive (≥1) or negative (<1). Dried blood spots were pre-diluted at a 1:40 dilution with 0.05% PBS-Tween using a Dynex Revelation automated absorbance microplate reader (Dynex Technologies; Chantilly, VA, USA). Plates were developed after 10 min using 3,3′,5,5′-tetramethylbenzidine core and orthophosphoric acid used as a stop solution (both TBS). Optical densities at 450 nm were measured using the Dynex Revelation. Results of ELISA for detection of anti-Spike antibodies in dried blood spot eluates have previously been shown to have almost perfect agreement with those performed on serum (Cohen’s kappa = 0.83)^18^.

### Sample size and statistical methods

Using the Stata powerlog programme, we estimated that a minimum sample size of 10,964 would be required to detect a difference of at least 2% in the proportion of exposed vs. unexposed participants experiencing a given binary outcome [equivalent to an odds ratio (OR) of 1.08], with 90% power, for a binary exposure with maximum variability (probability 0.50 changing to 0.52) and a moderate correlation (R2 = 0.4) with other variables in a logistic regression model, using a two-sided test and 5% significance. This post-vaccination symptom analysis was a pragmatic study including all participants meeting the inclusion criteria, with no sample size specified.

Logistic regression models were used to estimate odds ratios (ORs) and 95% confidence intervals (CIs) for associations between potential determinants of post-vaccination symptoms and their occurrence, first in a minimally adjusted, then fully adjusted models. Factors associating independently with each outcome at the *P* < 0.10 significance level were selected for inclusion in the multivariable model. Both the minimally adjusted and fully adjusted models were controlled for age (<30 years, 30 to <40 years, 40 to <50 years, 50 to <60 years, 60 to <70 years, and ≥70 years) and sex (male vs. female). Correction for multiple comparisons was not applied, on the grounds that we were testing a priori hypotheses for all risk factors investigated^20^. Unpaired two-sided *t* tests were used to compare mean anti-S titres and mean number of days from the date of second vaccine dose to the date of blood sampling between participants who did vs. did not experience reactive symptoms. Pearson chi-square tests were used to compare proportions of participants reporting post-vaccination symƒptoms by dose order and vaccine type. Linear regression was used in an exploratory analysis to evaluate determinants of the total number of systemic symptoms reported following a first dose of vaccine, and to test the association between frequency of reporting symptoms (never vs. after one vaccine dose only vs. after both vaccine doses) and mean post-vaccination antibody titres.

COVID-19 severity was classified into two groups: ‘asymptomatic’ (non-hospitalised seropositive participants, who either did not report any symptoms of acute respiratory infection or whose symptoms were classified as having <50% probability of being due to COVID-19, using the symptom algorithm by Menni and colleagues^21^) and ‘symptomatic’ (hospitalised and non-hospitalised seropositive participants who reported symptoms of acute respiratory infection that were classified as having ≥50% probability of being due to COVID-19, using the symptom algorithm.)^21^.

### Reporting summary

Further information on research design is available in the Nature Research Reporting Summary linked to this article.

## Supplementary information

SUPPLEMENTAL MATERIAL

REPORTING SUMMARY

## Data Availability

The data that support the findings of this study are available from the corresponding, Professor Adrian Martineau, author upon request. Project name is COVIDENCE UK.

## Code availability

The code written to implement statistical analyses for this study is available from h.holt@qmul.ac.uk upon reasonable request.

## References

[1] Johns Hopkins Coronavirus Resource Center. Coronavirus Resource Center. Available at https://coronavirus.jhu.edu2022.

[2] Sallam M (2021). COVID-19 vaccine hesitancy worldwide: a concise systematic review of vaccine acceptance rates. Vaccines.

[3] Herve C, Laupeze B, Del Giudice G, Didierlaurent AM, Tavares Da Silva F (2019). The how’s and what’s of vaccine reactogenicity. NPJ Vaccines..

[4] Rief W (2021). Fear of adverse effects and COVID-19 vaccine hesitancy: recommendations of the treatment expectation expert group. JAMA Health Forum.

[5] Chapin-Bardales J, Gee J, Myers T (2021). Reactogenicity following receipt of mRNA-Based COVID-19 Vaccines. J. Am. Med. Assoc..

[6] Menni C (2021). Vaccine side-effects and SARS-CoV-2 infection after vaccination in users of the COVID Symptom Study app in the UK: a prospective observational study. Lancet Infect. Dis..

[7] Coggins SA (2022). Adverse effects and antibody titers in response to the BNT162b2 mRNA COVID-19 vaccine in a prospective study of healthcare workers. Open Forum Infect. Dis.

[8] Naaber P (2021). Dynamics of antibody response to BNT162b2 vaccine after six months: a longitudinal prospective study. Lancet Reg. Health Eur..

[9] Krammer F (2021). Antibody responses in seropositive persons after a single dose of SARS-CoV-2 mRNA vaccine. N. Engl. J. Med..

[10] Hwang YH (2021). Can reactogenicity predict immunogenicity after COVID-19 vaccination?. Korean J. Intern. Med..

[11] Bauernfeind S (2021). Association between reactogenicity and immunogenicity after vaccination with BNT162b2. Vaccines (Basel)..

[12] Howren MB, Suls J (2011). The symptom perception hypothesis revised: depression and anxiety play different roles in concurrent and retrospective physical symptom reporting. J. Pers. Soc. Psychol..

[13] Calder PC (2022). Micronutrients to support vaccine immunogenicity and efficacy. Vaccines.

[14] Dickinson A., MacKay D. Health habits and other characteristics of dietary supplement users: a review. (1475–2891 (Electronic)). 10.1186/1475-2891-13-14 PMC3931917 24499096

[15] Bedford JL, Barr SI (2005). Diets and selected lifestyle practices of self-defined adult vegetarians from a population-based sample suggest they are more ‘health conscious’. Int. J. Behav. Nutr. Phys. Act..

[16] Talaei M (2022). Determinants of pre-vaccination antibody responses to SARS-CoV-2: a population-based longitudinal study (COVIDENCE UK). BMC Med..

[17] Jolliffe DA (2022). Determinants of antibody responses to SARS-CoV-2 vaccines: population-based longitudinal study (COVIDENCE UK). Vaccines (Basel).

[18] Cook AM (2021). Validation of a combined ELISA to detect IgG, IgA and IgM antibody responses to SARS-CoV-2 in mild or moderate non-hospitalised patients. J. Immunol. Methods.

[19] Holt H (2021). Risk factors for developing COVID-19: a population-based longitudinal study (COVIDENCE UK). Thorax.

[20] Rothman KJ (1990). No adjustments are needed for multiple comparisons. Epidemiology.

[21] Menni C (2020). Real-time tracking of self-reported symptoms to predict potential COVID-19. Nat. Med..

