# Supplementary material for: Incidence determinants and serological correlates of reactive symptoms following SARS-CoV-2 vaccination
Source: NPJ Vaccines. 2023 Feb 25;8:26. doi: 10.1038/s41541-023-00614-0 (PMC9959934; doi:10.1038/s41541-023-00614-0)
Supplement: Supplementary file 1 — SUPPLEMENTAL MATERIAL [file 41541_2023_614_MOESM1_ESM.docx]

**Incidence determinants and serological correlates of reactive symptoms following SARS-CoV-2 vaccination**

**Supplementary Material**

**Contents**

[**Supplementary Methods** 2](#_Toc125381396)

[Medication classification 2](#_Toc125381397)

[**Supplementary Tables** 4](#_Toc125381398)

[**Supplementary Table 1**. Baseline questions 4](#_Toc125381399)

[**Supplementary Table 2**. Follow-up questions 13](#_Toc125381400)

[**Supplementary Table 3.** Factors not associating with risk of systemic post-vaccination symptoms after adjustment for age and sex only 18](#_Toc125381401)

[**Supplementary Table 4.** Determinants of the number of systemic reactive symptoms after the first dose of SARS-CoV-2 vaccine among participants reporting at least one such symptom 19](#_Toc125381402)

[**Supplementary Table 5.** Incidence and determinants of systemic symptoms after a second dose of SARS-CoV-2 vaccine: minimally-adjusted and fully-adjusted analyses 20](#_Toc125381403)

[**Supplementary Table 6** Factors not associating with risk of local post-vaccination symptoms after adjustment for age and sex only 22](#_Toc125381404)

[**Supplementary Table 7.** Incidence and determinants of local symptoms after a second dose of SARS-CoV-2 vaccine: minimally-adjusted and fully adjusted analyses 23](#_Toc125381405)

[**Supplementary Table 8.** Post-vaccination antibody titres by vaccine type and post-vaccination reactive symptoms in subset of participants who did not have evidence of SARS-CoV-2 infection prior to vaccination1 25](#_Toc125381406)

[**Supplementary Table 9.** Association between self-reported long COVID and risk of systemic or local reactive symptoms following SARS-CoV-2 vaccination in in subset of participants who had serologic, antigen test or RT-PCR evidence of SARS-CoV-2 infection prior to vaccination. 26](#_Toc125381407)

[**Supplementary Figures** 27](#_Toc125381408)

[**Supplementary Figure 1.** Participant Flow 27](#_Toc125381409)

[**Supplementary Figure 2.** Pre-vaccination anti-S titres in participants who experienced symptomatic vs. asymptomatic SARS-CoV-2 infection 28](#_Toc125381410)

# **Supplementary Methods**

## Medication classification

The raw survey answers describing the names of medications prescribed to each participant were first processed using regular expressions to remove text related to dosage, route of administration, formulation, and frequency of administration. Each participant’s set of processed medication answers were then mapped to a curated, composite database containing 135,167 drug aliases (and their active ingredients) sourced from both the DrugBank and Electronic Medicines Compendium (EMC) databases. This mapping consisted of four steps. First, the composite database was searched for exact matches with each of the processed survey answers. For survey answers with no exact matches, the database was searched for an exact match with the first word of each survey answer. Then, the Metaphone algorithm ^7^ was applied to produce a phonetic encoding for each of the remaining unmapped survey answers and the entire composite drug database. Survey answers with an unambiguous, exact phonetic match with an alias in the database were annotated accordingly. Finally, for survey answers that could not be mapped in any of the three steps detailed above, Levenshtein distance (LD) ^8^ values were calculated between each unmapped survey answer and every alias in the drug database. For each processed survey answer, drug aliases in the database with an LD of 1 from the answer were identified. If the survey answer mapped to a single drug alias in the database with an LD of 1, the answer was annotated accordingly. If the survey answer mapped to multiple entries in the drug database with an LD of 1, the drug alias with the active ingredients appearing at the highest mean frequency across the entire annotated data set was selected. The remaining survey answers - for which no aliases in the database returned an LD value of 1 - were annotated manually.

With the active ingredient mapping for each survey answer, participant-level annotations were produced by considering each participant’s total set of survey answers. One set of covariates was produced by annotating each participant with a value of 1 for drug classes containing one or more compounds included in their survey answers and a value of 0 for all other drug classes. Another set of covariates was produced by annotating each participant with a scaled dosage value for each drug class, again taking a value of 0 for classes for which the participant did not report any medications. Z-score normalisation of dosages was applied separately for each active ingredient in each class. The dosage z-scores were subsequently transformed using a probit function, normalising them to values in the interval (0, 1) and allowing comparison between participants not taking any drugs from the class (dosage = 0) and participants taking varying dosages of drugs from the class (0 < dosage < 1). Code is available at [https://github.com/mgreenig/COVIDENCE-survey](https://eur01.safelinks.protection.outlook.com/?url=https%3A%2F%2Fgithub.com%2Fmgreenig%2FCOVIDENCE-survey&data=04%7C01%7C%7C62d495b62f724102264c08d8a423d2ca%7C569df091b01340e386eebd9cb9e25814%7C0%7C0%7C637439821203279159%7CUnknown%7CTWFpbGZsb3d8eyJWIjoiMC4wLjAwMDAiLCJQIjoiV2luMzIiLCJBTiI6Ik1haWwiLCJXVCI6Mn0%3D%7C1000&sdata=QWiOn%2Bl1kySG53HIdt1dy0rdOjNmDk0Et%2FEDlnTNMpA%3D&reserved=0)

# **Supplementary Tables**

## **Supplementary Table 1**. Baseline questions

| **Sociodemographic** | |
| --- | --- |
| Date of birth (DD/MM/YYYY) |  |
| Post code |  |
| Address |  |
| Please state your **assigned sex at birth.** | -Male  -Female |
| What is your ethnic origin? | - White   - English / Welsh / Scottish / Northern Irish / British - Irish - Gypsy or Irish Traveller - Any other white background   - Mixed / Multiple ethnic groups   - White and Black Caribbean - White and Black African - White and Asian - Any other Mixed / Multiple ethnic backgrounds   - Asian / Asian British   - Indian - Pakistani - Bangladeshi - Chinese - Any other Asian background   - Black / African / Caribbean / Black British   - African - Caribbean - Any other Black / African / Caribbean background   - Arab  - Other Ethnic Group |
| What is the highest level of education that you have completed? | - Primary school  - Secondary school up to 16 years  - Higher or secondary or further education (A-levels, BTEC, etc.)  - College or university  - Post-graduate degree |
| Which of the following best describes your current occupational status? | - Employed  - Self-employed  - Retired  - Furloughed  - Unemployed  - Not working due to sickness/ disability or illness  - Never employed  - Student  - Other |
| Please select the box that best describes your current housing situation: | - I own my home outright  - I own my home and I am paying a mortgage  - I am renting privately  - I am renting from the council/housing association  - I am staying with friends or family  - I am homeless or living in temporary accommodation  - Other |
| In the last month, was your household income sufficient to cover the basic needs of your household, such as food and heating? | - Yes  - Mostly  - Sometimes  - No |
| Do you currently claim Universal Credit? | - Yes, I have applied to receive Universal Credit but have not yet received any payments  - Yes, I have claimed Universal Credit and received one or more payments  - No |
| How many bedrooms are there in your current accommodation? | - 1  - 2  - 3  - 4  - 5  - 6  - 7  - 8  - 9  - 10 + |
| Do you live alone? | - Yes  - No |
| How many people other than yourself live in your household?  Children aged 0-4 years  Children aged 5-15 years  People aged 16-64 years  People aged 65 years or more | - 1  - 2  - 3  - 4  - 5  - 6  - 7  - 8  - 9  - 10 + |
| Does your household have any pets? | - Yes  - No |
| Please indicate which types of pet you have at home.  Select all that apply. | - Cat  - Dog  - Indoor bird (e.g. budgie, parrot, canary)  - Rabbit / Guinea pig / Hamster  - Tortoise, turtle, lizard or snake  - Other |
| **Behavioural** |  |
| In the last week, how many journeys did you make on public transport? (return trips count as 2 journeys) | - 1  - 2  - 3  - 4  - 5  - 6  - 7  - 8  - 9  - 10 + |
| In the last week, how many times have you been inside a shop or supermarket? | - 1  - 2  - 3  - 4  - 5  - 6  - 7  - 8  - 9  - 10 + |
| In the last week, how often have you been inside another indoor public space (e.g. café, pub, place of worship, restaurant, gym, day centre, waiting room, school, library, entertainment venue, hairdresser, takeaway restaurant)? | - 1  - 2  - 3  - 4  - 5  - 6  - 7  - 8  - 9  - 10 + |
| Over the last week, how many times have you been visited at home by someone who does not live in your household? (We are referring to visitors entering inside your home. People who just call at the door and do not come inside do not count) | - 1  - 2  - 3  - 4  - 5  - 6  - 7  - 8  - 9  - 10 + |
| Over the last week, how many times have you been into the home of someone who does not live in your household? (We are referring to visits in which you enter inside someone else’s home. Visits where you do not cross the threshold do not count. Neither do visits to the shops or other public places, we will ask about these later.) | - 1  - 2  - 3  - 4  - 5  - 6  - 7  - 8  - 9  - 10 + |
| Have you been advised by a doctor or other professional that you should be ‘SHIELDED’ during the coronavirus outbreak? (‘Shielding’ involves staying at home and minimising face-to-face contact with people outside the home. This advice has been given to people with certain underlying conditions that place them at increased risk of severe illness from COVID-19, including solid organ transplant recipients; people with specific cancers; people with severe respiratory conditions; people with severe combined immunodeficiency (SCID) or homozygous sickle cell disease; people on certain immunosuppressive treatments; and pregnant women with significant heart disease.) | - Yes  - No |
| In the last week, how frequently have you worn a face mask while in an indoor public place? | - Always (100% of the time)  - Usually (50-99% of the time)  - Sometimes (1-49% of the time)  - Never (0% of the time)  - Not applicable (I haven't been in a public place in the last week) |
| **Comorbidities** | |
| What is your **current** height?  (if you are unsure, please put your best estimate) | -Feet/inches  -Centimetres |
| What is your **current** weight? | - Stones (sts) / pounds (lbs)  - Kilograms (kg) |
| Have you ever been diagnosed with any of the following conditions by a doctor? Select all that apply | - Asthma  - Atopic Eczema or Atopic Dermatitis  - Autoimmune disease (e.g. rheumatoid arthritis, multiple sclerosis (MS), lupus (SLE), Crohn’s disease,  ulcerative colitis, psoriasis, Raynaud’s disease, scleroderma)  - Cancer  - Cerebral Palsy  - COPD (including chronic bronchitis, and emphysema)  - Cystic Fibrosis  - Dementia  - Diabetes or pre-diabetes  - Hayfever or Allergic Rhinitis  - Heart Attack, Angina or Coronary Artery Disease  - Heart Failure  - High Blood Pressure (Hypertension)  - HIV Infection  - Hyperparathyroidism (overactive parathyroid gland)  - Kidney stones  - Other kidney disease  - Leg Artery Disease (also known as ‘peripheral vascular disease’, ‘peripheral arterial disease’ or ‘intermittent claudication’)  - Mental health disorder  - Motor Neurone Disease  - Organ transplant  - Parkinson's Disease  - Primary immune deficiency (e.g. antibody deficiency, combined immunodeficiency)  - Sarcoidosis  - Sickle Cell Disease (i.e. two copies of altered gene, affected by anaemia and other complications  - Sickle Cell Carrier (also known as ‘sickle cell trait’, with only one copy of altered gene: few symptoms if any)  - Splenectomy (removal of spleen)  - Stroke or Mini-Stroke  - Tuberculosis (TB)  - None of the above |
| You indicated you have been diagnosed with diabetes or pre-diabetes. Please specify your diagnosis: | - Pre-diabetes (high blood sugar levels, not enough to be diagnosed with diabetes)  - Type 1 diabetes  - Type 2 diabetes  - Other type of diabetes |
| Do you currently have cancer? | - Never  - No, cancer cured or in remission  - Yes, currently receiving treatment |
| Under each heading, please click the ONE box that best describes your health TODAY.  Anxiety / Depression | - I am not anxious or depressed  - I am moderately anxious or depressed  - I am extremely anxious or depressed |
| Over the last 12 months, would you say that on the whole, your health has been: | - Excellent  - Very good  - Good  - Fair  - Poor |
| Are you currently pregnant? | - Yes  - No |
| What is your estimated date of delivery? (DD/MM/YYYY)  e.g. 25/04/2020 |  |
| Is this pregnancy a multiple pregnancy? (e.g. twins/triplets/etc.) | - Yes  - No |
| Have you experienced any of the following complications during this pregnancy? | - Pre-eclampsia/high blood pressure  - Gestational diabetes  - Other complication  - None of the above |
| How many pregnancies of 24 weeks or longer have you had, including this one? |  |
| How many pregnancies of less than 24 weeks have you had, including this one? |  |
| **Vaccination** | |
| Have you ever had the BCG vaccine?   *This is the vaccine against Tuberculosis (TB), it's injected in the upper arm and usually leaves a small scar* | - Yes  - No  - Unsure |
| Have you had a flu vaccine in the previous 12 months? | - Yes  - No |
| Have you had a pneumococcal vaccine in the previous 12 months? | - Yes  - No |
| Have you ever had the MMR vaccine?  This is the vaccine against measles, mumps and rubella. | - Yes  - No  -Unsure |
| Which COVID-19 vaccine did you have for your **FIRST dose**? | - Oxford / AstraZeneca / ChAdOx1  - Pfizer / BioNTech  - Moderna  -Novavax  -Valneva  - Janssen (also known as Johnson & Johnson)  -Other – please specify  - Not sure / don’t know |
| On what **date** did you have your **first** vaccine dose? If you are not sure of the exact date, enter the approximate date (DD/MM/YYYY) e.g. for 16th December 2020, write 16/12/2020 |  |
| At what **time of day** did you have your **first** vaccine dose? (We are asking this question as there is emerging evidence that time of day can affect vaccine responses. We appreciate that this information may be difficult to recall, especially if you were vaccinated a while back: if you are not sure, either give your best guess or click ‘I don’t remember’) | - Morning (before 12 pm)  - Lunchtime (12 pm to 2 pm)  - Afternoon (2 pm to 5 pm)  - Evening (5 pm onwards)  - I don’t remember |
| Did you experience any of the following symptoms following your first dose of COVID-19 vaccine? Click as many as apply. | - Tenderness, soreness, swelling, redness or a painful heavy feeling at the injection site  - Feeling tired  - Headache  - Fever / high temperature (37.8° C or greater)  - Muscle aches  - Swelling of the glands in your armpit or neck  -Other symptoms: specify  -None of the above |
| On what **date** did you have your **second** vaccine dose? If you are not sure of the exact date, enter the approximate date (DD/MM/YYYY) e.g. for 16th December 2020, write 16/12/2020 |  |
| At what **time of day** did you have your **second** vaccine dose? (We are asking this question as there is emerging evidence that time of day can affect vaccine responses. We appreciate that this information may be difficult to recall, especially if you were vaccinated a while back: if you are not sure, either give your best guess or click ‘I don’t remember’) | - Morning (before 12 pm)  - Lunchtime (12 pm to 2 pm)  - Afternoon (2 pm to 5 pm)  - Evening (5 pm onwards)  - I don’t remember |
| Did you experience any of the following symptoms following your second dose of COVID-19 vaccine? Click as many as apply. | - Tenderness, soreness, swelling, redness or a painful heavy feeling at the injection site  - Feeling tired  - Headache  - Fever / high temperature (37.8° C or greater)  - Muscle aches  - Swelling of the glands in your armpit or neck  -Other symptoms: specify  -None of the above |
| **Lifestyle** | |
| Which of these best describes your use of cigarettes? | - I have never smoked cigarettes  - I used to smoke cigarettes occasionally but now not at all  - I used to smoke cigarettes daily but now not at all  - I smoke cigarettes occasionally but not every day  - I smoke cigarettes daily |
| Which of these best describes your use of e-cigarettes (vaping)? | - I have never vaped or used e-cigarettes  - I used to use e-cigarettes occasionally, but now not at all  - I used to use e-cigarettes daily but now not at all  - I vape occasionally but not every day  - I vape daily |
| During the last week, roughly how many hours did you spend doing more vigorous physical exercise of sufficient intensity to make you breathless or to raise your heart rate significantly, such as heavy physical work, more strenuous gardening (e.g. vigorous digging, landscaping) swimming, jogging, aerobics, football, tennis, cycling, gym workout? | - 0 -10+ hours |
| During the last week, roughly how many hours did you spend doing light exercise that does not make you particularly breathless, such as light gardening, walking, including walking for pleasure or exercise, walking to the shops, walking to work? | - 0 -10+ hours |
| During the past month, how many hours of actual sleep did you get per night on average?  (This may be different than the number of hours you spent in bed) | - 0 -24 hours |
| **Diet** | |
| Do you **exclude** any of the following foods from your diet? Select all that apply. | - Eggs  - Cow’s milk or products made from cow’s milk (e.g. cheese, yoghurt)  - Fish  - White meat (e.g. poultry)  - Red meat  - No, I eat all of these foods |
| How many units of alcohol did you drink over the last 7 days?    One unit is a ½ a pint (285 ml) of ordinary beer, lager or cider; 25ml of spirits; 1 small glass (75ml) of wine; or 50ml of sherry. | - None  - 1-7 units  - 8-14 units  - 15-21 units  - 22-28 units  - More than 28 units |
| Over the **last month**, have you taken any of the following supplements at least **once per week**?  Select all that apply. | - Multivitamin (including prenatal multivitamins)  - Supplement containing vitamin A only  - Supplement containing vitamin B only  - Supplement containing vitamin C only  - Supplement containing vitamin D only  - Supplement containing calcium only  - Supplement containing calcium and vitamin D combined  - Supplement containing vitamin E only  - Supplement containing zinc only  - Supplement containing iron only  - Supplement containing probiotics  - Supplement containing fish oil, krill oil or other source of omega-3 fatty acids  - Supplement containing cod liver oil  - Supplement containing echinacea  - Supplement containing garlic or garlic powder (allicin)  - Supplement containing turmeric / curcumin  - Supplement containing Cannabidiol (CBD) oil  - Supplements containing folic acid  - Supplement containing Selenium only  - Other (e.g. other micronutrients (such as herbal supplements) or combinations of micronutrients (such vitamin C & zinc)) Please specify:  - None of the above |
| **Medications** | |
| Please type the names of all the medications you are currently taking below, one medication per box. The next pages will collect details about dosage for each.   Include all types of medications taken at home or administered in a hospital or clinic (capsules, tablets, contraceptive pills or implants, inhalers, injections, intravenous infusions, monoclonal antibodies, chemotherapy, immunosuppressants, etc.)   Please note that other pages will collect details about the amount of medicine in each dose (next page) and how often you take each dose (the page after that). If there are any details about your medication that aren’t captured by our form (e.g. if you take different doses of a medicine at different times of day), there will be space to enter them in a blank text box at the end of this section of the questionnaire. | 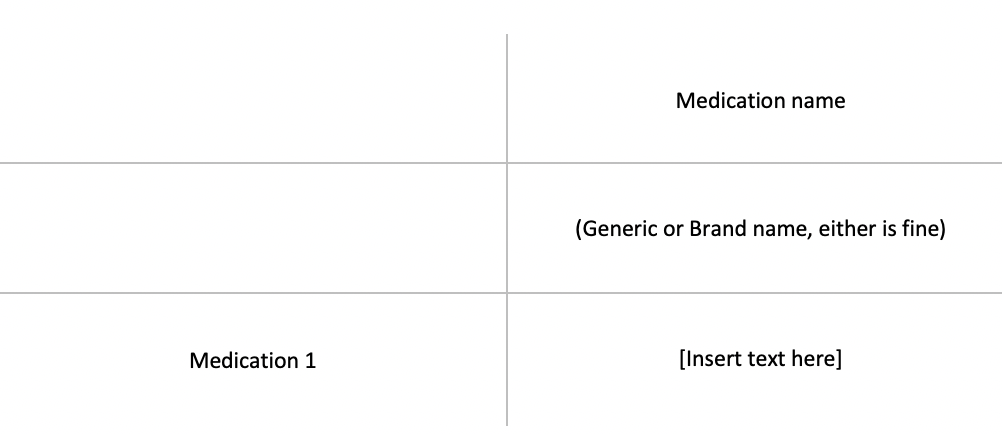 |
| Please select the frequency and route that you take your medication: | - Frequency   - 4 times/day - 3 times/day - 2 times/day - Once daily - Weekly - Less often than weekly - As needed   - Route   - By mouth - Inhaled - Injected - Other |
| **Quality of life** |  |
| First, have you or your family noticed that you are currently experiencing any of the following problems?  Please answer all of these questions WHETHER OR NOT YOU HAVE HAD DEFINITE OR SUSPECTED COVID-19.  Excessive shortness of breath  Coughing  Unusual tiredness or fatigue  Problems with sleeping  Memory problems  Difficulty concentrating  Pains in your muscles or joints  Problems with your sense of smell / taste  Diarrhoea  Stomach (abdominal) pains  Changes to your voice  Hair loss  Unusual racing of the heart  Lightheadedness or dizziness  Unusual sweating | -No  -Yes but improving  -Yes, but not improving or worsening  -Yes and worsening |
| Second, here is a list of statements relating to common long-term symptoms of COVID-19. Please select one answer per line to indicate how you have been feeling over the past 7 days.  Please answer all of these questions WHETHER OR NOT YOU HAVE HAD DEFINITE OR SUSPECTED COVID-19.  I feel fatigued  I feel weak all over  I feel listless ("washed out")  I feel tired  I have trouble starting things because I am tired  I have trouble finishing things because I am tired  I have energy  I am able to do my usual activities  I need to sleep during the day  I am too tired to eat  I need help doing my usual activities  I am frustrated by being too tired to do the things I want to do  I have to limit my social activity because I am tired | -Not at all  -A little bit  -Somewhat  -Quite a bit  -Very much |
| Third, would YOU say that you currently have 'long COVID', i.e. ongoing symptoms more than four weeks after the onset of proven or suspected COVID-19? | - Yes  - No  -Don’t know / not sure |
| By selecting one box in each group below, please indicate which statements best describe your own health state today. |  |
| Self-Care | - I have no problems with self-care  - I have some problems washing or dressing myself  - I am unable to wash or dress myself |
| Mobility | - I have no problems in walking about  - I have some problems in walking about  - I am confined to bed |
| Usual Activities (e.g. work, study, housework, family or leisure activities) | - I have no problems with performing my usual activities  - I have some problems with performing my usual activities  - I am unable to perform my usual activities |
| Pain / Discomfort | - I have no pain or discomfort  - I have moderate pain or discomfort  - I have extreme pain or discomfort |
| Anxiety / Depression | - I am not anxious or depressed  - I am moderately anxious or depressed  - I am extremely anxious or depressed |
| In the last week, how often have you been bothered by the following problems?  Little interest or pleasure in doing things  Feeling down, depressed, or hopeless  Feeling nervous, anxious or on edge  Not being able to stop or control worrying | - Not at all  - Several days (1-3 days/week)  - More than half the days (4-5 days/week)  - Nearly every day (6-7 days/week) |
| Over the last month, would you say that on the whole, your health has been: | - Excellent  - Very good  - Good  - Fair  - Poor |
| **Recent health** | |
| Since February 1st 2020, have you experienced any of the following symptoms: loss of smell or taste, fever, persistent cough, fatigue, diarrhoea, abdominal pain or loss of appetite? | - Yes, I have had one or more of these symptoms since 1st of February  - No, I have not had any of these symptoms since 1st of February |
| When did your symptoms start? (DD/MM/YYYY) *e.g. 25/04/2020* |  |
| Did you have a persistent cough (coughing a lot for more than an hour, or 3 or more coughing episodes in 24 hours)? | - No  - Persistent dry cough (i.e. producing little or no phlegm)  - Persistent productive cough |
| Did you experience unusual fatigue? | - No  - Mild fatigue  - Severe fatigue - I struggled to get out of bed |
| Did you have a loss of sense of smell or taste? | - Yes  - No |
| Did you skip any meals because you felt unwell? | - Yes  - No |
| Since February 1st 2020, have you had a nose/throat swab to test for COVID-19? | - Yes  - No |
| On what date did you have this nose/throat swab?  If you are not sure of the exact date, enter the approximate date (DD/MM/YYYY).  *e.g. 25/04/2020* |  |
| What was the result? | - Positive  - Negative  - Not known |
| Since you last checked in with us, have you had an attack (exacerbation) of asthma or COPD (chronic bronchitis / emphysema)? | - Yes  - No |
| Was this an attack of asthma or COPD? | - Asthma attack  - COPD attack |
| Did you go to hospital for treatment of your asthma/COPD attack? | - No, I didn’t go to hospital for treatment of my attack  - Yes, I attended a hospital accident and emergency department but I was not admitted to hospital (i.e. I went home without spending a night in hospital)  - Yes, and I was admitted to hospital (i.e. I spent one or more nights in hospital) |
| On what date did you attend hospital? Please select a date on the calendar below.  If you are not sure of the exact date, click on the approximate date. |  |
| Did this asthma/COPD attack require treatment with steroid tablets (e.g. prednisolone)? | - Yes  - No |
| Did this asthma/COPD attack require treatment with antibiotics (e.g. amoxycillin, clarithromycin, augmentin)? | - Yes  - No  -Don’t know / not sure |

## **Supplementary Table 2**. Follow-up questions

| **Questions asked at every monthly follow-up** |  |
| --- | --- |
| **Sociodemographic** |  |
| Which of the following best describes your current occupational status? | - Employed  - Self-employed  - Retired  - Furloughed  - Unemployed  - Not working due to sickness/ disability or illness  - Never employed  - Student  - Other |
| Please select the box that best describes your current housing situation: | - I own my home outright  - I own my home and I am paying a mortgage  - I am renting privately  - I am renting from the council/housing association  - I am staying with friends or family  - I am homeless or living in temporary accommodation  - Other |
| In the last month, was your household income sufficient to cover the basic needs of your household, such as food and heating? | - Yes  - Mostly  - Sometimes  - No |
| Do you currently claim Universal Credit? | - Yes, I have applied to receive Universal Credit but have not yet received any payments  - Yes, I have claimed Universal Credit and received one or more payments  - No |
| **Behavioural** |  |
| In the last week, how many journeys did you make on public transport? (return trips count as 2 journeys) | - 1  - 2  - 3  - 4  - 5  - 6  - 7  - 8  - 9  - 10 + |
| In the last week, how many times have you been inside a shop or supermarket? | - 1  - 2  - 3  - 4  - 5  - 6  - 7  - 8  - 9  - 10 + |
| In the last week, how often have you been inside another indoor public space (e.g. café, pub, place of worship, restaurant, gym, day centre, waiting room, school, library, entertainment venue, hairdresser, takeaway restaurant)? | - 1  - 2  - 3  - 4  - 5  - 6  - 7  - 8  - 9  - 10 + |
| Over the last week, how many times have you been visited at home by someone who does not live in your household? (We are referring to visitors entering inside your home. People who just call at the door and do not come inside do not count) | - 1  - 2  - 3  - 4  - 5  - 6  - 7  - 8  - 9  - 10 + |
| Over the last week, how many times have you been into the home of someone who does not live in your household? (We are referring to visits in which you enter inside someone else’s home. Visits where you do not cross the threshold do not count. Neither do visits to the shops or other public places, we will ask about these later.) | - 1  - 2  - 3  - 4  - 5  - 6  - 7  - 8  - 9  - 10 + |
| Have you been advised by a doctor or other professional that you should be ‘SHIELDED’ during the coronavirus outbreak? (‘Shielding’ involves staying at home and minimising face-to-face contact with people outside the home. This advice has been given to people with certain underlying conditions that place them at increased risk of severe illness from COVID-19, including solid organ transplant recipients; people with specific cancers; people with severe respiratory conditions; people with severe combined immunodeficiency (SCID) or homozygous sickle cell disease; people on certain immunosuppressive treatments; and pregnant women with significant heart disease.) | - Yes  - No |
| In the last week, how frequently have you worn a face mask while in an indoor public place? | - Always (100% of the time)  - Usually (50-99% of the time)  - Sometimes (1-49% of the time)  - Never (0% of the time)  - Not applicable (I haven't been in a public place in the last week) |
| **Comorbidities** |  |
| Under each heading, please click the ONE box that best describes your health TODAY.  Anxiety / Depression | - I am not anxious or depressed  - I am moderately anxious or depressed  - I am extremely anxious or depressed |
| Over the last 12 months, would you say that on the whole, your health has been: | - Excellent  - Very good  - Good  - Fair  - Poor |
| **Vaccinations** |  |
| Since you last checked in with us, have you had a flu jab (vaccine against influenza)? | - Yes  - No |
| On what date did you have this flu jab (immunisation)? Please select a date on the calendar below.  If you are not sure of the exact date, click on the approximate date. |  |
| Which COVID-19 vaccine did you have for your **FIRST dose**? | - Oxford / AstraZeneca / ChAdOx1  - Pfizer / BioNTech  - Moderna  -Novavax  -Valneva  - Janssen (also known as Johnson & Johnson)  -Other – please specify  - Not sure / don’t know |
| On what **date** did you have your **first** vaccine dose? If you are not sure of the exact date, enter the approximate date (DD/MM/YYYY) e.g. for 16th December 2020, write 16/12/2020 |  |
| At what **time of day** did you have your **first** vaccine dose? (We are asking this question as there is emerging evidence that time of day can affect vaccine responses. We appreciate that this information may be difficult to recall, especially if you were vaccinated a while back: if you are not sure, either give your best guess or click ‘I don’t remember’) | - Morning (before 12 pm)  - Lunchtime (12 pm to 2 pm)  - Afternoon (2 pm to 5 pm)  - Evening (5 pm onwards)  - I don’t remember |
| Did you experience any of the following symptoms following your first dose of COVID-19 vaccine? Click as many as apply. | - Tenderness, soreness, swelling, redness or a painful heavy feeling at the injection site  - Feeling tired  - Headache  - Fever / high temperature (37.8° C or greater)  - Muscle aches  - Swelling of the glands in your armpit or neck  -Other symptoms: specify  -None of the above |
| On what **date** did you have your **second** vaccine dose? If you are not sure of the exact date, enter the approximate date (DD/MM/YYYY) e.g. for 16th December 2020, write 16/12/2020 |  |
| At what **time of day** did you have your **second** vaccine dose? (We are asking this question as there is emerging evidence that time of day can affect vaccine responses. We appreciate that this information may be difficult to recall, especially if you were vaccinated a while back: if you are not sure, either give your best guess or click ‘I don’t remember’) | - Morning (before 12 pm)  - Lunchtime (12 pm to 2 pm)  - Afternoon (2 pm to 5 pm)  - Evening (5 pm onwards)  - I don’t remember |
| Did you experience any of the following symptoms following your second dose of COVID-19 vaccine? Click as many as apply. | - Tenderness, soreness, swelling, redness or a painful heavy feeling at the injection site  - Feeling tired  - Headache  - Fever / high temperature (37.8° C or greater)  - Muscle aches  - Swelling of the glands in your armpit or neck  -Other symptoms: specify  -None of the above |
| **Lifestyle** |  |
| Which of these best describes your use of cigarettes? | - I have never smoked cigarettes  - I used to smoke cigarettes occasionally but now not at all  - I used to smoke cigarettes daily but now not at all  - I smoke cigarettes occasionally but not every day  - I smoke cigarettes daily |
| Which of these best describes your use of e-cigarettes (vaping)? | - I have never vaped or used e-cigarettes  - I used to use e-cigarettes occasionally, but now not at all  - I used to use e-cigarettes daily but now not at all  - I vape occasionally but not every day  - I vape daily |
| During the last week, roughly how many hours did you spend doing more vigorous physical exercise of sufficient intensity to make you breathless or to raise your heart rate significantly, such as heavy physical work, more strenuous gardening (e.g. vigorous digging, landscaping) swimming, jogging, aerobics, football, tennis, cycling, gym workout? | - 0 -10+ hours |
| During the last week, roughly how many hours did you spend doing light exercise that does not make you particularly breathless, such as light gardening, walking, including walking for pleasure or exercise, walking to the shops, walking to work? | - 0 -10+ hours |
| During the past month, how many hours of actual sleep did you get per night on average?  (This may be different than the number of hours you spent in bed) | - 0 -24 hours |
| **Diet** |  |
| Over the **last month**, have you taken any of the following supplements at least **once per week**?  Select all that apply. | - Multivitamin (including prenatal multivitamins)  - Supplement containing vitamin A only  - Supplement containing vitamin B only  - Supplement containing vitamin C only  - Supplement containing vitamin D only  - Supplement containing calcium only  - Supplement containing calcium and vitamin D combined  - Supplement containing vitamin E only  - Supplement containing zinc only  - Supplement containing iron only  - Supplement containing probiotics  - Supplement containing fish oil, krill oil or other source of omega-3 fatty acids  - Supplement containing cod liver oil  - Supplement containing echinacea  - Supplement containing garlic or garlic powder (allicin)  - Supplement containing turmeric / curcumin  - Supplement containing Cannabidiol (CBD) oil  - Supplements containing folic acid  - Supplement containing Selenium only  - Other (e.g. other micronutrients (such as herbal supplements) or combinations of micronutrients (such vitamin C & zinc)) Please specify:  - None of the above |
| **Quality of life** |  |
| First, have you or your family noticed that you are currently experiencing any of the following problems?  Please answer all of these questions WHETHER OR NOT YOU HAVE HAD DEFINITE OR SUSPECTED COVID-19.  Excessive shortness of breath  Coughing  Unusual tiredness or fatigue  Problems with sleeping  Memory problems  Difficulty concentrating  Pains in your muscles or joints  Problems with your sense of smell / taste  Diarrhoea  Stomach (abdominal) pains  Changes to your voice  Hair loss  Unusual racing of the heart  Lightheadedness or dizziness  Unusual sweating | -No  -Yes but improving  -Yes, but not improving or worsening  -Yes and worsening |
| Second, here is a list of statements relating to common long-term symptoms of COVID-19. Please select one answer per line to indicate how you have been feeling over the past 7 days.  Please answer all of these questions WHETHER OR NOT YOU HAVE HAD DEFINITE OR SUSPECTED COVID-19.  I feel fatigued  I feel weak all over  I feel listless ("washed out")  I feel tired  I have trouble starting things because I am tired  I have trouble finishing things because I am tired  I have energy  I am able to do my usual activities  I need to sleep during the day  I am too tired to eat  I need help doing my usual activities  I am frustrated by being too tired to do the things I want to do  I have to limit my social activity because I am tired | -Not at all  -A little bit  -Somewhat  -Quite a bit  -Very much |
| Third, would YOU say that you currently have 'long COVID', i.e. ongoing symptoms more than four weeks after the onset of proven or suspected COVID-19? | - Yes  - No  -Don’t know / not sure |
| By selecting one box in each group below, please indicate which statements best describe your own health state today. |  |
| Self-Care | - I have no problems with self-care  - I have some problems washing or dressing myself  - I am unable to wash or dress myself |
| Mobility | - I have no problems in walking about  - I have some problems in walking about  - I am confined to bed |
| Usual Activities (e.g. work, study, housework, family or leisure activities) | - I have no problems with performing my usual activities  - I have some problems with performing my usual activities  - I am unable to perform my usual activities |
| Pain / Discomfort | - I have no pain or discomfort  - I have moderate pain or discomfort  - I have extreme pain or discomfort |
| Anxiety / Depression | - I am not anxious or depressed  - I am moderately anxious or depressed  - I am extremely anxious or depressed |
| In the last week, how often have you been bothered by the following problems?  Little interest or pleasure in doing things  Feeling down, depressed, or hopeless  Feeling nervous, anxious or on edge  Not being able to stop or control worrying | - Not at all  - Several days (1-3 days/week)  - More than half the days (4-5 days/week)  - Nearly every day (6-7 days/week) |
| **Recent Health** |  |
| Since you last checked in with us, have you had a nose or throat swab for COVID-19 or any other respiratory virus, or has a result from a previous swab test become newly available?(This question is about tests to detect the virus itself: they are usually done in somebody who has symptoms, but screening of asymptomatic people can also be done. It’s usually a nose/throat swab, but saliva tests are also becoming available) | - Yes  - No |
| On what date did you have this nose / throat swab? If you are not sure of the exact date, enter the approximate date (DD/MM/YYYY). |  |
| What was the result? Click as many as apply. | - Positive for COVID-19 (SARS-CoV-2 coronavirus)  - Positive for influenza virus  - Positive for another respiratory virus  - Negative for all/any viruses tested  - Not Known |
| Since you last checked in with us, have you experienced any of the following symptoms: cold or flu symptoms, sore throat, persistent cough, loss of smell or taste, fever, fatigue, diarrhoea, abdominal pain or loss of appetite? | - Yes, I have had one or more of these symptoms since completing my last COVIDENCE UK questionnaire  - No, I have not had any of these symptoms since completing my last COVIDENCE UK questionnaire |
| When did your symptoms start? (DD/MM/YYYY) |  |
| Did you have a persistent cough (coughing a lot for more than an hour, or 3 or more coughing episodes in 24 hours)? | - No  - Persistent dry cough (i.e. producing little or no phlegm)  - Persistent productive cough |
| Did you experience unusual fatigue? | - No  - Mild fatigue  - Severe fatigue - I struggled to get out of bed |
| Did you have a loss of sense of smell or taste? | - Yes  - No |
| Did you skip any meals because you felt unwell? | - Yes  - No |
| Since you last checked in with us, have you had an attack (exacerbation) of asthma or COPD (chronic bronchitis / emphysema)? | - Yes  - No |
| Was this an attack of asthma or COPD? | - Asthma attack  - COPD attack |
| Did you go to hospital for treatment of your asthma/COPD attack? | - No, I didn’t go to hospital for treatment of my attack  - Yes, I attended a hospital accident and emergency department but I was not admitted to hospital (i.e. I went home without spending a night in hospital)  - Yes, and I was admitted to hospital (i.e. I spent one or more nights in hospital) |
| On what date did you attend hospital? Please select a date on the calendar below.  If you are not sure of the exact date, click on the approximate date. |  |
| Did this asthma/COPD attack require treatment with steroid tablets (e.g. prednisolone)? | - Yes  - No |
| Did this asthma/COPD attack require treatment with antibiotics (e.g. amoxycillin, clarithromycin, augmentin)? | - Yes  - No  -Don’t know / not sure |
| Since you last checked in with us, have you had any other serious illness or injury?    i.e. something that did NOT present with loss of smell or taste, fever, persistent cough, fatigue, diarrhoea, abdominal pain or loss of appetite or an attack (exacerbation) of asthma or COPD symptoms.    By serious, we mean an illness or injury:  - that was life-threatening, or  - that caused you to be admitted to hospital, or  - that prolonged your hospital stay (if you were already in hospital when it happened), or  - that resulted in a lasting disability or permanent damage to your health  We ONLY need to know about serious medical events you have experienced since you last checked in with us, i.e. just those which fulfill the criteria above. If you had an illness or injury that does not fullfill the criteria above, click ‘No’ below to move to the next question. | - Yes, I have one or more other serious illnesses or injuries since my last questionnaire  - No, I have not had any other serious illnesses or injuries since my last questionnaire |
| How many other serious illnesses or injuries have you had since you last checked in with us? |  |
| Please name and briefly describe the nature of any other serious illnesses / injuries that you have experienced since your last questionnaire here (e.g. heart attack, stroke).    If you have more than one illness or injury to report, put each one in a separate box. |  |
| Please describe the nature of the other serious illnesses/injuries that you have experienced since your last questionnaire here. | Was this illness/injury life threatening?  - Yes  - No  Were you admitted to hospital for this illness/injury?  - Yes  - No |
| Please describe the nature of the other serious illnesses/injuries that you have experienced since your last questionnaire here. | If you were already in hospital when you experienced this illness or injury, did it prolong your hospital stay?  - Yes  - No  - N/A  Did this illness or injury result in a lasting disability or permanent damage to your health?  - Yes  - No |

## **Supplementary Table 3.** Factors not associating with risk of systemic post-vaccination symptoms after adjustment for age and sex only

|  |  | **After first dose** | | | **After second dose** | | |
| --- | --- | --- | --- | --- | --- | --- | --- |
|  |  | **N (%) symptomatic** | **Adjusted OR**  **(95% CI)** | **P** | **N (%) symptomatic** | **Adjusted OR**  **(95% CI)** | **P** |
| **Non-SSRI antidepressants** | No | 4,078/8,624 (47.3) | 1.00 |  | 2,401/8,624 (27.8) | 1.00 |  |
|  | Yes | 184/379 (48.6) | 0.96 (0.77-1.18) | 0.67 | 121/379 (31.9) | 1.12 (0.89-1.40) | 0.323 |
| **ARBs** | No | 4,008/8,443 (47.5) | 1.00 |  | 2,387/8,443 (28.3) | 1.00 |  |
|  | Yes | 254/560 (45.4) | 1.05 (0.88-1.25) | 0.58 | 135/560 (24.1) | 1 .94 (0.77-1.16) | 0.58 |
| **Vitamin K antagonists** | No | 4,243/8,955 (47.4) | 1.00 |  | 2,508/8,955 (28.0) | 1.00 |  |
|  | Yes | 19/48 (39.6) | 0.93 (0.51-1.69) | 0.81 | 14/48 (29.2) | 1.38 (0.73-2.60) | 0.33 |
| **Beta-blockers** | No | 3,982/8,359 (47.7) | 1.00 |  | 2,348/8,359 (28.1) | 1.00 |  |
|  | Yes | 280/644 (43.5) | 1.01 (0.85-1.19) | 0.95 | 174/644 (27.0) | 1.15 (0.95-1.38) | 0.15 |
| **Thiazides** | No | 4,136/8,708 (47.5) | 1.00 |  | 2,458/8,708 (28.2) | 1.00 |  |
|  | Yes | 126/295 (42.7) | 0.93 (0.73-1.18) | 0.55 | 64/295 (21.7) | 0.82 (0.61-1.09) | 0.16 |
| **H2-receptor antagonists** | No | 4,228/8,939 (47.3) | 1.00 |  | 2,501/8,939 (28.0) | 1.00 |  |
|  | Yes | 34/64 (53.1) | 1.27 (0.77-2.09) | 0.36 | 21/64 (32.8) | 1.28 (0.75-2.18) | 0.36 |
| **Calcium channel blockers** | No | 3,876/8,088 (47.9) | 1.00 |  | 2,296/8,088 (28.4) | 1.00 |  |
|  | Yes | 386/915 (42.2) | 0.95 (0.82-1.09) | 0.46 | 226/915 (24.7) | 1.02 (0.87-1.20) | 0.82 |
| **SGLT2 inhibitors** | No | 4,240/8,955 (47.4) | 1.00 |  | 2,507/8,955 (28.0) | 1.00 |  |
|  | Yes | 22/48 (45.8) | 1.06 (0.59-1.89) | 0.84 | 15/48 (31.3) | 1.37 (0.74-2.55) | 0.32 |
| **Anti-platelet drugs** | No | 4,006/8,404 (47.7) | 1.00 |  | 2,371/8,404 (28.2) | 1.00 |  |
|  | Yes | 256/599 (42.7) | 1.05 (0.88-1.25) | 0.59 | 151/599 (25.2) | 1.12 (0.92-1.36) | 0.26 |
| **Sex hormone therapy** | No | 3,877/8,301 (46.7) | 1.00 |  | 2,284/8,301 (27.5) | 1.00 |  |
|  | Yes | 385/702 (54.8) | 1.07 (0.91-1.25) | 0.43 | 238/702 (33.9) | 1.07 (0.90-1.26) | 0.45 |
| **Metformin** | No | 4,162/8,750 (47.6) | 1.00 |  | 2,453/8,750 (28.0) | 1.00 |  |
|  | Yes | 100/253 (39.5) | 0.84 (0.65-1.10) | 0.20 | 69/253 (27.3) | 1.15 (0.87-1.54) | 0.33 |
| **Bisphosphonates** | No | 4,184/8,841 (47.3) | 1.00 |  | 2,482/8,841 (28.1) | 1.00 |  |
|  | Yes | 78/162 (48.2) | 0.99 (0.72-1.36) | 0.95 | 40/162 (24.7) | 0.84 (0.58-1.21) | 0.34 |
| **Aspirin** | No | 4,052/8,531 (47.5) | 1.00 |  | 2,402/8,531 (28.2) | 1.00 |  |
|  | Yes | 210/472 (44.5) | 1.14 (0.94-1.38) | 0.20 | 120/472 (25.4) | 1.13 (0.91-1.41) | 0.27 |
| **BCG vaccinated** | No | 499/1,087 (45.9) | 1.00 |  | 316/1,087 (29.1) | 1.00 |  |
|  | Yes | 3,416/7,084 (48.2) | 1.01 (0.89-1.16) | 0.83 | 2,008/7,084 (28.4) | 0.90 (0.78-1.04) | 0.15 |

## **Supplementary Table 4.** Determinants of the number of systemic reactive symptoms after the first dose of SARS-CoV-2 vaccine among participants reporting at least one such symptom

|  |  | **N** | **Mean no. of systemic symptoms reported (s.d.)** | **Adjusted mean difference (95% CI)^1^** | **P-value** |
| --- | --- | --- | --- | --- | --- |
| **Vaccine type** | ChAdOx1 | 2,324 | 1.69 (0.89) | -- (Ref) | -- |
|  | BNT162b2 | 564 | 1.20 (0.52) | -0.50 (-0.58 to -0.43) | <0.001 |
|  | MRNA-1273 | 1 | 1 (--) | -0.62 (-2.26 to 1.01) | 0.456 |
|  | Other | 22 | 1.73 (0.94) | -0.01 (-0.34 to 0.36) | 0.940 |
| **Month of dose** | Q1 (Jan-Mar) | 2,809 | 1.60 (0.85) | 0.09 (-0.09 to 0.27) | 0.306 |
|  | Q2 (Apr-Jun) | 93 | 1.61 (0.89) | -- (Ref) | -- |
|  | Q3 (Jul-Sep) | 0 | N/A | N/A | -- |
|  | Q4 (Oct-Dec) | 20 | 1.10 (0.45) | -0.06 (-0.47 to 0.34) | 0.771 |
| **Age, years** | 16-29.99 | 16 | 2.00 (1.21) | -- (Ref) |  |
|  | 30-39.99 | 50 | 1.52 (0.79) | -0.42 (-0.89 to 0.05) | 0.079 |
|  | 40-49.99 | 196 | 1.69 (0.94) | -0.34 (-0.77 to 0.08) | 0.112 |
|  | 50-59.99 | 808 | 1.66 (0.89) | -0.41 (-0.82 to 0.01) | 0.054 |
|  | 60-69.99 | 1,267 | 1.58 (0.83) | -0.46 (-0.87 to -0.05) | 0.027 |
|  | ≥70.00 | 587 | 1.53 (0.80) | -0.48 (-0.89 to -0.06) | 0.024 |
|  | P for trend |  |  |  | 0.010 |
| **Sex** | Female | 2,172 | 1.62 (0.87) | -- (Ref) |  |
|  | Male | 752 | 1.54 (0.80) | -0.08 (-0.15 to -0.01) | 0.028 |
| **Self-rated general health** | Excellent | 592 | 1.60 (0.87) | -- (Ref) |  |
|  | Very good | 1,156 | 1.59 (0.83) | -0.01 (-0.09 to 0.07) | 0.814 |
|  | Good | 770 | 1.54 (0.81) | -0.05 (-0.14 to 0.05) | 0.335 |
|  | Fair | 317 | 1.67 (0.91) | 0.08 (-0.04 to 0.19) | 0.212 |
|  | Poor | 89 | 1.84 (1.05) | 0.22 (0.03 to 0.41) | 0.026 |
|  | P for trend |  |  |  | 0.147 |
| **Pre-vaccination SARS-CoV-2 status** | Seronegative | 2,485 | 1.58 (0.84) | -- (Ref) |  |
|  | Seropositive asymptomatic | 304 | 1.65 (0.91) | 0.70 (-0.03 to 0.17) | 0.165 |
|  | Seropositive symptomatic | 135 | 1.76 (0.93) | 0.18 (0.04 to 0.33) | 0.013 |
|  | P for trend |  |  |  | 0.006 |
| **Self-rated anxiety or depression** | No | 2,156 | 1.58 (0.84) | -- (Ref) |  |
|  | Yes | 766 | 1.64 (0.88) | 0.05 (-0.03 to 0.12) | 0.213 |
| **Atopic disease^2^** | No | 2,134 | 1.58 (0.83) | -- (Ref) |  |
|  | Yes | 790 | 1.66 (0.91) | 0.08 (0.01 to 0.15) | 0.019 |
| **Arterial disease** | No | 2,766 | 1.60 (0.85) | -- (Ref) |  |
|  | Yes | 158 | 1.56 (0.92) | -0.02 (-0.17 to 0.12) | 0.767 |
| **Kidney disease** | No | 2,862 | 1.60 (0.85) | -- (Ref) |  |
|  | Yes | 62 | 1.73 (0.93) | 0.12 (-0.09 to 0.33) | 0.258 |
| **Cancer** | Never | 2,642 | 1.60 (0.86) | -- (Ref) |  |
|  | Previous | 261 | 1.57 (0.84) | -0.00 (-0.11 to 0.10) | 0.949 |
|  | Active | 21 | 1.43 (0.75) | -0.08 (-0.44 to 0.28) | 0.654 |
| **Statins** | No | 2,439 | 1.61 (0.86) | -- (Ref) |  |
|  | Yes | 485 | 1.54 (0.82) | -0.03 (-0.12 to 0.06) | 0.548 |
| **ACE inhibitors** | No | 2,658 | 1.59 (0.85) | -- (Ref) |  |
|  | Yes | 266 | 1.66 (0.91) | -0.09 (-0.02 to 0.20) | 0.093 |
| **Multivitamin supplement** | No | 2,273 | 1.60 (0.85) | -- (Ref) |  |
|  | Yes | 651 | 1.61 (0.86) | 0.02 (-0.06 to 0.9) | 0.659 |

1, mutually adjusted for all covariates presented in the table

2, atopic disease defined by self-report of atopic eczema/dermatitis and/or hayfever/allergic rhinitis

## **Supplementary Table 5.** Incidence and determinants of systemic symptoms after a second dose of SARS-CoV-2 vaccine: minimally-adjusted and fully-adjusted analyses

|  |  |  | **Minimally-adjusted** | | **Fully-adjusted** | |
| --- | --- | --- | --- | --- | --- | --- |
|  |  | **N (%) symptomatic** | **aOR (95% CI)** | **P** | **aOR (95% CI)** | **P** |
| **Vaccine type** | Oxford / AstraZeneca | 1,668/5,988 (27.9) | 1.00 |  | 1.00 |  |
|  | Pfizer / BioNTech | 827/2,864 (28.8) | 1.32 (1.02-1.25) | 0.017 | 1.13 (1.02-1.26) | 0.023 |
|  | Moderna | 5/59 (8.5) | 0.22 (0.09-0.56) | 0.002 | 0.88 (0.30-2.53) | 0.82 |
|  | Other | 22/92 (23.9) | 0.79 (0.48-1.28) | 0.34 | 0.78 (0.46-1.31) | 0.34 |
| **Month of dose** | Q1 (Jan-Mar) | 233/751 (30.0) | 1.14 (0.97-1.35) | 0.11 | 1.34 (1.10-1.64) | 0.004 |
|  | Q2 (Apr-Jun) | 2,283/7,975 (28.3) | 1.00 |  | 1.00 |  |
|  | Q3 (Jul-Sep) | 3/274 (1.09) | -- | -- | -- | -- |
|  | Q4 (Oct-Dec) | 3/3 (100) | -- | -- | -- | -- |
| **Inter-dose interval** | <6 weeks | 86/326 (26.4) | 0.86 (0.66-1.11) | 0.24 | 0.61 (0.46-0.83) | 0.001 |
|  | 6-10 weeks | 676/2,522 (26.8) | 0.83 (0.74-0.92) | 0.001 | 0.86 (0.76-0.96) | 0.009 |
|  | >10 weeks | 1,760/6,155 (28.6) | 1.00 |  | 1.00 |  |
|  | P for trend |  |  |  | (1.20, 1.09-1.33) | <0.001 |
| **Age, years** | 16-29.99 | 34/120 (28.3) | 1.00 |  | 1.00 |  |
|  | 30-39.99 | 92/341 (27.0) | 0.94 (0.59-1.49) | 0.78 | 0.82 (0.48-1.38) | 0.45 |
|  | 40-49.99 | 246/800 (30.8) | 1.10 (0.72-1.68) | 0.67 | 0.68 (0.42-1.09) | 0.11 |
|  | 50-59.99 | 795/2,192 (36.3) | 1.43 (0.95-2.14) | 0.09 | 0.77 (0.48-1.22) | 0.26 |
|  | 60-69.99 | 958/3,617 (26.5) | 0.94 (0.62-1.41) | 0.75 | 0.51 (0.32-0.80) | 0.004 |
|  | ≥70.00 | 397/1,933 (20.5) | 0.71 (0.47-1.08) | 0.11 | 0.37 (0.23-0.59) | <0.001 |
|  | P for trend |  |  |  | 0.80 (0.76-0.83) | <0.001 |
| **Sex** | Female | 1,997/6,367 (31.4) | 1.00 |  | 1.00 |  |
|  | Male | 525/2,636 (19.9) | 0.59 (0.53-0.66) | <0.001 | 0.63 (0.56-0.71) | <0.001 |
| **Vaping status** | Not current vaper | 2,438/8,778 (27.8) | 1.00 |  | 1.00 |  |
|  | Current vaper | 78/207 (37.7) | 1.47 (1.10-1.97) | 0.009 | 1.29 (0.96-1.76) | 0.09 |
| **Alcohol, units/wk** | 0 | 759/2,349 (32.3) | 1.00 |  | 1.00 |  |
|  | 1-7 | 902/3,189 (28.3) | 0.84 (0.75-0.95) | 0.005 | 0.90 (0.80-1.02) | 0.09 |
|  | 8-14 | 496/1,853 (26.8) | 0.82 (0.71-0.94) | 0.004 | 0.92 (0.80-1.06) | 0.28 |
|  | 15-21 | 217/901 (24.1) | 0.73 (0.61-0.87) | <0.001 | 0.78 (0.65-0.94) | 0.010 |
|  | 22-28 | 84/403 (20.8) | 0.64 (0.49-0.82) | 0.001 | 0.69 (0.53-0.90) | 0.006 |
|  | >28 | 64/308 (20.8) | 0.64 (0.48-0.86) | 0.003 | 0.66 (0.49-0.89) | 0.007 |
|  | P for trend |  |  |  | 0.93 (0.89-0.96) | <0.001 |
| **Sleep, hr/night** | ≤6 | 227/765 (29.7) | 1.08 (0.91-1.29) | 0.36 | 0.89 (0.74-1.06) | 0.20 |
|  | 7 | 672/2,163 (31.1) | 1.21 (1.07-1.36) | 0.002 | 1.13 (1.00-1.27) | 0.05 |
|  | 8 | 997/3,706 (26.9) | 1.00 |  | 1.00 |  |
|  | ≥9 | 625/2,365 (26.4) | 0.97 (0.86-1.09) | 0.63 | 0.98 (0.87-1.11) | 0.81 |
| **Self-rated general health** | Excellent | 392/1,878 (20.9) | 1.00 |  | 1.00 |  |
|  | Very good | 950/3,590 (26.5) | 1.41 (1.23-1.61) | <0.001 | 1.36 (1.18-1.56) | <0.001 |
|  | Good | 723/2,326 (31.1) | 1.74 (1.51-2.01) | <0.001 | 1.55 (1.34-1.81) | <0.001 |
|  | Fair | 346/949 (36.5) | 2.13 (1.79-2.53) | <0.001 | 1.71 (1.41-2.08) | <0.001 |
|  | Poor | 111/260 (42.7) | 2.69 (2.05-3.54) | <0.001 | 1.95 (1.43-2.64) | <0.001 |
|  | P for trend |  |  |  | 1.19 (1.12-1.25) | <0.001 |
| **Pre-vaccination SARS-CoV-2 status** | Seronegative | 2,102/7,640 (27.5) | 1.00 |  | 1.00 |  |
|  | Seropositive asymptomatic | 255/958 (26.6) | 0.97 (0.83, 1.13) | 0.661 | 0.97 (0.83-1.14) | 0.741 |
|  | Seropositive symptomatic | 165/405 (40.7) | 1.58 (1.26-1.97) | <0.001 | 1.68 (1.35-2.09) | <0.001 |
|  | P for trend |  |  |  | 1.18 (1.08-1.30) | <0.001 |
| **Self-rated anxiety or depression** | No | 1,727/6,744 (25.6) | 1.00 |  | 1.00 |  |
|  | Yes | 792/2,253 (35.2) | 1.45 (1.31-1.61) | <0.001 | 1.27 (1.13-1.42) | <0.001 |
| **Asthma** | No | 2,035/7,552 (27.0) | 1.00 |  | 1.00 |  |
|  | Yes | 487/1,451 (33.6) | 1.31 (1.16-1.48) | <0.001 | 1.11 (0.94-1.32) | 0.23 |
| **Atopic disease^2^** | No | 1,772/6,684 (26.5) | 1.00 |  | 1.00 |  |
|  | Yes | 750/2,319 (32.3) | 1.26 (1.13-1.40) | <0.001 | 1.15 (1.03-1.28) | 0.015 |
| **COPD** | No | 2,464/8,830 (27.90) | 1.00 |  | 1.00 |  |
|  | Yes | 58/173 (33.5) | 1.52 (1.10-2.11) | 0.011 | 1.23 (0.8- 1.76) | 0.25 |
| **Heart disease** | No | 2,423/8,642 (28.0) | 1.00 |  | 1.00 |  |
|  | Yes | 99/361 (27.4) | 1.32 (1.03-1.68) | 0.028 | 0.96 (0.63-1.47) | 0.86 |
| **Arterial disease** | No | 2,386/8,514 (28.0) | 1.00 |  | 1.00 |  |
|  | Yes | 136/489 (27.8) | 1.33 (1.08-1.65) | 0.008 | 1.19 (0.78-1.80) | 0.43 |
| **Kidney disease** | No | 2,461/8,821 (27.9) | 1.00 |  | 1.00 |  |
|  | Yes | 61/182 (33.5) | 1.49 (1.08-2.04) | 0.014 | 1.21 (0.88-1.68) | 0.24 |
| **Major neurological condition** | No | 2,448/8,752 (28.0) | 1.00 |  | -- | -- |
|  | Yes | 74/251 (29.5) | 1.29 (0.97-1.70) | 0.08 | 0.97 (0.66-1.44) | 0.90 |
| **Immunodeficiency** | No | 2,502/8,950 (28.0) | 1.00 |  | 1.00 |  |
|  | Yes | 20/53 (37.7) | 1.65 (0.94-2.91) | 0.08 | 1.23 (0.68-2.22) | 0.49 |
| **Proton pump inhibitors** | No | 2,155/7,752 (27.8) | 1.00 |  | 1.00 |  |
|  | Yes | 367/1,251 (29.3) | 1.20 (1.05-1.37) | 0.008 | 0.94 (0.81-1.09) | 0.41 |
| **Inhaled corticosteroids** | No | 2,313/8,415 (27.5) | 1.00 |  | 1.00 |  |
|  | Yes | 209/588 (35.5) | 1.43 (1.20-1.71) | <0.001 | 1.21 (0.95-1.55) | 0.13 |
| **Systemic immunosuppressants** | No | 2,389/8,607 (27.8) | 1.00 |  | 1.00 |  |
|  | Yes | 133/396 (33.6) | 0.33 (1.07-1.65) | 0.010 | 1.02 (0.81-1.29) | 0.86 |
| **SSRIs** | No | 2,321/8,430 (27.5) | 1.00 |  | 1.00 |  |
|  | Yes | 201/573 (35.1) | 1.26 (1.05-1.50) | 0.014 | 0.98 (0.81-1.19) | 0.84 |
| **Inhaled bronchodilators** | No | 2,228/8,166 (27.3) | 1.00 |  | 1.00 |  |
|  | Yes | 294/837 (35.1) | 1.41 (1.21-1.64) | <0.001 | 1.23 (0.47-3.19) | 0.67 |
| **Paracetamol (chronic use)** | No | 2,393/8,627 (27.7) | 1.00 |  | 1.00 |  |
|  | Yes | 129/376 (34.3) | 1.45 (1.16-1.81) | 0.001 | 1.17 (0.92-1.48) | 0.19 |
| **Beta-2 adrenergic agonists** | No | 2,235/8,192 (27.3) | 1.00 |  | 1.00 |  |
|  | Yes | 287/811 (35.4) | 1.41 (1.21-1.65) | <0.001 | 0.84 (0.33-2.16) | 0.72 |
| **Anticholinergics** | No | 2,384/8,591 (27.8) | 1.00 |  | 1.00 |  |
|  | Yes | 138/412 (33.5) | 1.27 (1.03-1.57) | 0.028 | 0.73 (0.53-0.99) | 0.045 |
| **Multivitamin supplement** | No | 1,923/7,168 (26.8) | 1.00 |  | 1.00 |  |
|  | Yes | 599/1,835 (32.6) | 1.27 (1.14-1.42) | <0.001 | 1.27 (1.13-1.43) | <0.001 |
| **Vitamin D supplement** | No | 1,897/6,690 (28.4) | 1.00 |  | 1.00 |  |
|  | Yes | 625/2,313 (27.0) | 0.89 (0.80-1.00) | 0.043 | 0.92 (0.82-1.02) | 0.12 |

## **Supplementary Table 6** Factors not associating with risk of local post-vaccination symptoms after adjustment for age and sex only

|  |  | **N (%) symptomatic** | **Adjusted OR**  **(95% CI)** | **P** | **N (%) symptomatic** | **Adjusted OR**  **(95% CI)** | **P** |
| --- | --- | --- | --- | --- | --- | --- | --- |
| **ACE inhibitors** | No | 3,553/8,101 (43.9) | 1.00 |  | 2,657/8,101 (32.8) | 1.00 |  |
|  | Yes | 361/902 (40.1) | 1.06 (0.92-1.23) | 0.41 | 263/902 (29.2) | 1.05 (0.90-1.23) | 0.53 |
| **Systemic immunosuppressants** | No | 3,737/8,607 (43.4) | 1.00 |  | 2,788/8,607 (32.4) | 1.00 |  |
|  | Yes | 177/396 (44.7) | 1.06 (0.86-1.30) | 0.59 | 132/396 (33.3) | 1.05 (0.84-1.30) | 0.67 |
| **ARBs** | No | 3,692/8,443 (43.7) | 1.00 |  | 2,771/8,443 (32.8) | 1.00 |  |
|  | Yes | 222/560 (39.6) | 1.00 (0.84-1.20) | 0.96 | 149/560 (26.6) | 0.88 (0.72-1.07) | 0.19 |
| **Vitamin K antagonists** | No | 3,898/8,955 (43.5) | 1.00 |  | 2,910/8,955 (32.5) | 1.00 |  |
|  | Yes | 16/48 (33.3) | 0.87 (0.47-1.61) | 0.65 | 10/48 (20.8) | 0.72 (0.35-1.48) | 0.38 |
| **Beta-blockers** | No | 3,651/8,359 (43.7) | 1.00 |  | 2,742/8,359 (32.8) | 1.00 |  |
|  | Yes | 263/644 (40.8) | 1.11 (0.93-1.31) | 0.25 | 178/644 (27.6) | 0.96 (0.80-1.16) | 0.69 |
| **Calcium channel blockers** | No | 3,544/8,088 (43.8) | 1.00 |  | 2,668/8,088 (33.0) | 1.00 |  |
|  | Yes | 370/915 (40.4) | 1.11 (0.96-1.28) | 0.17 | 252/915 (27.5) | 0.97 (0.83-1.14) | 0.75 |
| **Metformin** | No | 3,811/8,750 (43.6) | 1.00 |  | 2,845/8,750 (32.5) | 1.00 |  |
|  | Yes | 103/253 (40.7) | 1.10 (0.85-1.44) | 0.46 | 75/253 (29.6) | 1.09 (0.82-1.44) | 0.57 |
| **Bisphosphonates** | No | 3,843/8,841 (43.5) | 1.00 |  | 2,863/8,841 (32.4) | 1.00 |  |
|  | Yes | 71/162 (43.5) | 0.97 (0.71-1.34) | 0.87 | 57/162 (35.2) | 1.10 (0.79-1.52) | 0.59 |

## **Supplementary Table 7.** Incidence and determinants of local symptoms after a second dose of SARS-CoV-2 vaccine: minimally-adjusted and fully adjusted analyses

|  |  |  | **Minimally-adjusted** | | **Fully-adjusted** | |
| --- | --- | --- | --- | --- | --- | --- |
|  |  | **N (%) symptomatic** | **aOR (95% CI)** | **P** | **aOR (95% CI)** | **P** |
| **Vaccine type** | Oxford / AstraZeneca | 1,639/5,988 (27.4) | 1.00 |  | 1.00 |  |
|  | Pfizer / BioNTech | 1,252/2,864 (43.7) | 2.39 (2.17-2.64) | <0.001 | 2.57 (2.3- 2.85) | <0.001 |
|  | Moderna | 5/59 (8.47) | 0.25 (0.10-0.63) | 0.003 | 0.84 (0.29-2.44) | 0.75 |
|  | Other | 24/92 (26.1) | 0.93 (0.57-1.49) | 0.75 | 0.80 (0.48-1.33) | 0.39 |
| **Month of dose** | Q1 (Jan-Mar) | 287/751 (38.2) | 1.33 (1.13-1.56) | <0.001 | 0.90 (0.76-1.07) | 0.70 |
|  | Q2 (Apr-Jun) | 2,628/7,975 (33.0) | 1.00 |  | 1.00 |  |
|  | Q3 (Jul-Sep) | 2/274 (0.73) | 0.01 (0.00-0.04) | <0.001 | 0.08 (0.00, 0.03) | <0.001 |
|  | Q4 (Oct-Dec) | 3/3 (100) | -- |  | -- |  |
| **Inter-dose interval** | <6 weeks | 101/326 (31.0) | 0.92 (0.72-1.18) | 0.51 | -- | -- |
|  | 6-10 weeks | 820/2,522 (32.5) | 0.92 (0.83-1.02) | 0.12 | -- | -- |
|  | >10 weeks | 1,999/6,155 (32.5) | 1.00 |  | -- | -- |
|  | P for trend |  |  |  | -- | -- |
| **Age, years** | 16-29.99 | 42/120 (35.0) | 1.00 |  | 1.00 |  |
|  | 30-39.99 | 96/341 (28.2) | 0.73 (0.46-1.14) | 0.16 | 0.66 (0.38-1.12) | 0.12 |
|  | 40-49.99 | 289/800 (36.1) | 1.02 (0.68-1.53) | 0.93 | 0.74 (0.46-1.20 | 0.23 |
|  | 50-59.99 | 882/2,192 (40.2) | 1.23 (0.84-1.82) | 0.29 | 0.80 (0.47-1.21) | 0.34 |
|  | 60-69.99 | 1,177/3,617 (32.5) | 0.93 (0.63-1.36) | 0.70 | 0.59 (0.37-0.93) | 0.025 |
|  | ≥70.00 | 434/1,933 (22.5) | 0.60 (0.40-0.89) | 0.011 | 0.34 (0.21-0.56) | <0.001 |
|  | P for trend |  |  |  | 0.80 (0.76-0.84) | <0.001 |
| **Sex** | Female | 2,358/6,367 (37.0) | 1.00 |  | 1.00 |  |
|  | Male | 562/2,636 (21.3) | 0.50 (0.45-0.55) | <0.001 | 0.50 (0.44-0.56) | <0.001 |
| **Ethnicity** | Asian/Asian British | 28/94 (29.8) | 0.86 (0.55-1.36) | 0.52 | -- | -- |
|  | Black/Black British | 13/36 (36.1) | 0.94 (0.47-1.88) | 0.86 | -- | -- |
|  | Mixed/other | 65/197 (33.0) | 0.96 (0.71-1.30) | 0.78 | -- | -- |
|  | White | 2,814/8,676 (32.4) | 1.00 |  | -- | -- |
| **Education** | Primary/Secondary | 282/978 (28.8) | 0.81 (0.68-0.95) | 0.009 | 0.75 (0.63-0.89) | 0.001 |
|  | Higher/Further | 400/1,265 (31.6) | 0.87 (0.75-1.00) | 0.05 | 0.86 (0.74-1.00) | 0.05 |
|  | College | 1,270/3,991 (31.8) | 0.88 (0.80-0.98) | 0.020 | 0.87 (0.78-0.97) | 0.010 |
|  | Post-graduate | 965/2,762 (34.9) | 1.00 |  | 1.00 |  |
|  | P for trend |  |  |  | 1.09 (1.04-1.15) | 0.001 |
| **Alcohol, units/wk** | 0 | 810/2,349 (34.5) | 1.00 |  | 1.00 |  |
|  | 1-7 | 1,083/3,189 (34.0) | 1.00 (0.89-1.12) | 0.98 | 1.04 (0.92-1.17) | 0.57 |
|  | 8-14 | 568/1,853 (30.7) | 0.90 (0.79-1.03) | 0.14 | 0.97 (0.85-1.12) | 0.71 |
|  | 15-21 | 251/901 (27.9) | 0.82 (0.69-0.97) | 0.022 | 0.87 (0.73-1.04) | 0.13 |
|  | 22-28 | 106/403 (26.3) | 0.81 (0.63-1.03) | 0.09 | 0.84 (0.65-1.09) | 0.19 |
|  | >28 | 102/308 (33.1) | 1.17 (0.90-1.52) | 0.24 | 1.22 (0.93-1.60) | 0.15 |
|  | P for trend |  |  |  | 0.99 (0.95-1.02) | 0.44 |
| **Light physical exercise, hr/wk** | 0-4 | 975/2,832 (34.4) | 1.18 (1.06-1.33) | 0.003 | 1.09 (0.97-1.23) | 0.15 |
|  | 5-9 | 1,010/2,983 (33.9) | 1.15 (1.03,-1.29) | 0.011 | 1.12 (1.00-1.25) | 0.06 |
|  | ≥10 | 932/3,172 (29.4) | 1.00 |  | 1.00 |  |
|  | P for trend |  |  |  | 0.96 (0.90-1.02) | 0.14 |
| **Dietary restrictions** | None | 2,733/8,500 (32.2) | 1.00 |  | 1.00 |  |
|  | Vegetarian | 154/397 (38.8) | 1.22 (0.99-1.51) | 0.06 | 1.23 (0.99-1.54) | 0.09 |
|  | Vegan | 2,733/8,500 (32.2) | 0.87 (0.57 to 1.33) | 0.52 | 1.00 (0.62 to 1.51) | 0.89 |
| **Self-rated general health** | Excellent | 542/1,878 (28.9) | 1.00 |  | 1.00 |  |
|  | Very good | 1,105/3,590 (30.8) | 1.13 (1.00-1.28) | 0.05 | 1.10 (0.96-1.25) | 0.17 |
|  | Good | 822/2,326 (35.3) | 1.37 ( 1.20-1.56) | <0.001 | 1.24 (1.07-1.43) | 0.004 |
|  | Fair | 348/949 (36.7) | 1.38 (1.17-1.64) | <0.001 | 1.13 (0.94-1.37) | 0.20 |
|  | Poor | 103/260 (39.6) | 1.53 (1.16-2.01) | 0.002 | 1.16 (0.86-1.57) | 0.34 |
|  | P for trend |  |  |  | 1.06 (1.00-1.11) | 0.039 |
| **Pre-vaccination SARS-CoV-2 status** | Seronegative | 2,425/7,640 (31.7) | 1.00 |  | 1.00 |  |
|  | Seropositive asymptomatic | 320/958 (33.4) | 1.10 (0.95-1.27) | 0.19 | 1.15 (0.99-1.33) | 0.08 |
|  | Seropositive symptomatic | 175/405 (43.2) | 1.54 (1.23-1.91) | <0.001 | 1.54 (1.24-1.93) | <0.001 |
|  | P for trend |  |  |  | 1.21 (1.10-1.33) | <0.001 |
| **Self-rated anxiety or depression** | No | 2,071/6,744 (30.7) | 1.00 |  | 1.00 |  |
|  | Yes | 848/2,253 (37.6) | 1.24 (1.12-1.37) | <0.001 | 1.16 (1.03-1.30) | 0.013 |
| **Asthma** | No | 2,366/7,552 (31.3) | 1.00 |  | 1.00 |  |
|  | Yes | 554/1,451 (38.2) | 1.29 (1.15-1.46) | <0.001 | 1.16 (0.98-1.37) | 0.08 |
| **Atopic disease^1^** | No | 2,068/6,684 (30.9) | 1.00 |  | 1.00 |  |
|  | Yes | 852/2,319 (36.7) | 1.23 (1.11-1.36) | <0.001 | 1.15 (1.03-1.28) | 0.016 |
| **Arterial disease** | No | 2,777/8,514 (32.6) | 1.00 |  | 1.00 |  |
|  | Yes | 143/489 (29.2) | 1.20 (0.98-1.48) | 0.08 | 0.83 (0.61-1.12) | 0.22 |
| **Major neurological condition** | No | 2,837/8,752 (32.4) | 1.00 |  | 1.00 |  |
|  | Yes | 83/251 (33.1) | 1.28 (0.97-1.68) | 0.08 | 1.20 (0.85-1.70) | 0.30 |
| **Cancer** | Never | 2,666/8,117 (32.8) | 1.00 |  | 1.00 |  |
|  | Previous | 228/809 (28.2) | 0.85 (0.72-1.00) | 0.05 | 0.83 (0.70-0.99) | 0.033 |
|  | Active | 26/77 (33.8) | 1.41 (0.87-2.31) | 0.17 | 1.09 (0.66-1.82) | 0.74 |
| **Immunodeficiency** | No | 2,897/8,950 (32.4) | 1.00 |  | 1.00 |  |
|  | Yes | 23/53 (43.4) | 1.75 (1.00-3.05) | 0.05 | 1.38 (0.77-2.47) | 0.28 |
| **Statins** | No | 2,455/7,377 (33.3) | 1.00 |  | 1.00 |  |
|  | Yes | 465/1,626 (28.6) | 1.15 (1.01-1.31) | 0.032 | 0.99 (0.86-1.15) | 0.89 |
| **Proton pump inhibitors** | No | 2,487/7,752 (32.1) | 1.00 |  | 1.00 |  |
|  | Yes | 433/1,251 (34.6) | 1.26 (1.11-1.44) | <0.001 | 1.08 (0.94-1.25) | 0.29 |
| **Inhaled corticosteroids** | No | 2,693/8,415 (32.0) | 1.00 |  | 1.00 |  |
|  | Yes | 227/588 (38.6) | 1.30 (1.09-1.55) | 0.003 | 1.00 (0.78-1.28) | 0.99 |
| **SSRIs** | No | 2,692/8,430 (31.9) | 1.00 |  | 1.00 |  |
|  | Yes | 228/573 (39.8) | 1.23 (1.03-1.47) | 0.023 | 1.05 (0.87-1.28) | 0.61 |
| **H2-receptor antagonists** | No | 2,887/8,939 (32.3) | 1.00 |  | 1.00 |  |
|  | Yes | 33/64 (51.6) | 2.31 (1.40-3.83) | 0.001 | 2.05 (1.21-3.47) | 0.008 |
| **Inhaled bronchodilators** | No | 2,228/8,166 (27.3) | 1.00 |  | 1.00 |  |
|  | Yes | 294/837 (35.1) | 1.41 (1.21-1.64) | <0.001 | 1.23 (0.47-3.19) | 0.67 |
| **Anti-platelet drugs** | No | 2,724/8,404 (32.4) | 1.00 |  | 1.00 |  |
|  | Yes | 196/599 (32.7) | 1.42 (1.18-1.71) | <0.001 | 1.13 (0.70-1.83) | 0.60 |
| **Sex hormone therapy** | No | 2,627/8,301 (31.7) | 1.00 |  | 1.00 |  |
|  | Yes | 293/702 (41.7) | 1.18 (1.00-1.39) | 0.044 | 1.14 (0.96-1.35) | 0.12 |
| **Paracetamol (chronic use)** | No | 2,393/8,627 (27.7) | 1.00 |  | 1.00 |  |
|  | Yes | 129/376 (34.3) | 1.45 (1.16-1.81) | 0.001 | 1.17 (0.92-1.48) | 0.19 |
| **Beta-2 adrenergic agonists** | No | 2,235/8,192 (27.3) | 1.00 |  | 1.00 |  |
|  | Yes | 287/811 (35.4) | 1.41 (1.21-1.65) | <0.001 | 0.84 (0.33-2.16) | 0.72 |
| **Anticholinergics** | No | 2,384/8,591 (27.8) | 1.00 |  | 1.00 |  |
|  | Yes | 138/412 (33.5) | 1.27 (1.03-1.57) | 0.028 | 0.73 (0.53-0.99) | 0.045 |
| **Aspirin** | No | 2,763/8,531 (32.4) | 1.00 |  | 1.00 |  |
|  | Yes | 157/472 (33.3) | 1.45 (1.18-1.78) | <0.001 | 1.26 (0.77-2.04) | 0.35 |
| **Multivitamin supplement** | No | 1,923/7,168 (26.8) | 1.00 |  | 1.00 |  |
|  | Yes | 599/1,835 (32.6) | 1.27 (1.14-1.42) | <0.001 | 1.27 (1.13-1.43) | <0.001 |
| **Vitamin D supplement** | No | 1,897/6,690 (28.4) | 1.00 |  | 1.00 |  |
|  | Yes | 625/2,313 (27.0) | 0.89 (0.80-1.00) | 0.043 | 0.92 (0.82-1.02) | 0.12 |

## **Supplementary Table 8.** Post-vaccination antibody titres by vaccine type and post-vaccination reactive symptoms in subset of participants who did not have evidence of SARS-CoV-2 infection prior to vaccination1

|  | | | Post-vaccination antibody titres | | | |
| --- | --- | --- | --- | --- | --- | --- |
|  | N | Mean titre (s.d.) | Mean difference (95% CI) | P | Coefficient (95% CI) | P for trend |
| **ChAdOx1** | | | | | | |
| Local reactive symptoms |  |  |  |  |  |  |
| Never | 1,846 | 2.35 (1.58) | -- (ref) | -- | 0.12, 0.06 to 0.19 per increasing category | <0.001 |
| After one dose only | 1,028 | 2.42 (1.54) | 0.06 (-0.05 to 0.17) | 0.275 |  |  |
| After both doses | 814 | 2.61 (1.58) | 0.26 (0.13 to 0.39) | <0.001 |  |  |
| Systemic reactive symptoms |  |  |  |  |  |  |
| Never | 1,226 | 2.28 (1.57) | -- (ref) | -- | 0.16, 0.09 to 0.22, per increasing category | <0.001 |
| After one dose only | 1,528 | 2.44 (1.53) | 0.16 (0.04 to 0.28) | 0.008 |  |  |
| After both doses | 934 | 2.59 (1.64) | 0.31 (0.18 to 0.44) | <0.001 |  |  |
| **BNT162b2** | | | | | | |
| Local reactive symptoms |  |  |  |  |  |  |
| Never | 613 | 4.11 (2.60) | -- (ref) | -- | 0.14, 0.06 to 0.28, per increasing category | 0.041 |
| After one dose only | 426 | 4.45 (2.62) | 0.34 (0.03 to 0.65) | 0.034 |  |  |
| After both doses | 682 | 4.40 (2.44) | 0.29 (0.02 to 57) | 0.038 |  |  |
| Systemic reactive symptoms |  |  |  |  |  |  |
| Never | 969 | 4.16 (2.48) | -- (ref) | -- | 0.26, 0.10 to 0.41, per increasing category | 0.002 |
| After one dose only | 467 | 4.39 (2.52) | 0.23 (-0.05 to 0.51) | 0.105 |  |  |
| After both doses | 285 | 4.68 (2.76) | 0.52 (0.19 to 0.86) | 0.002 |  |  |

1, i.e. excluding participants who were anti-S sero-positive and/or who reported a positive RT-PCR or lateral flow swab test result for SARS-CoV-2, prior to vaccination

## **Supplementary Table 9.** Association between self-reported long COVID and risk of systemic or local reactive symptoms following SARS-CoV-2 vaccination in in subset of participants who had serologic, antigen test or RT-PCR evidence of SARS-CoV-2 infection prior to vaccination.

|  |  | **First dose** | | | | | **Second dose** | | | | |
| --- | --- | --- | --- | --- | --- | --- | --- | --- | --- | --- | --- |
|  |  | N (%) symptomatic | Minimally adjusted aOR (95% CI)^1^ | P-value | Fully adjusted OR (95% CI) | P-value | N (%) symptomatic | Minimally adjusted OR (95% CI)^1^ | P- value | Fully adjusted OR (95% CI) | P-value |
| **Systemic symptoms** | | | | | | | | | | | |
| Long COVID | No | 431 (45.6) | -- (ref) |  | -- (ref) |  | 248 (26.3) | -- (ref) |  | -- (ref) |  |
|  | Yes | 140 (67.0) | 2.40 (1.71 to 3.35) | <0.001 | 1.73 (1.12 to 2.65)^2^ | 0.013 | 90 (43.1) | 2.02 (1.46 to 2.81) | <0.001 | 1.26 (0.83 to 1.90)^3^ | 0.273 |
| **Local symptoms** | | | | | | | | | | | |
| Long COVID | No | 404 (42.8) | -- (ref) |  | -- (ref) |  | 312 (33.1) | -- (ref) |  | -- (ref) |  |
|  | Yes | 128 (62.2) | 1.94 (1.41 to 2.66) | <0.001 | 1.74 (1.07 to 2.82)^4^ | 0.025 | 96 (45.9) | 1.64 (1.20 to 2.25) | 0.002 | 1.41 (0.93 to 2.12)^5^ | 0.104 |

1, adjusted for age and sex only

2, adjusted for age, sex, IMD quartile rank, anxiety/depression, alcohol intake, cigarette use, pre-vaccination SARS-CoV-2 infection status, asthma, atopy, arterial disease, kidney disease, cancer, self-assessed general health, vaccine type, quarter of first dose, multivitamin use, vitamin D use, beta-2 adrenergic agonists, statins, ACE inhibitors, inhaled corticosteroids, inhaled bronchodilators, immunosuppressants, long covid status

3, adjusted for age, sex, sleep per night, anxiety/depression, alcohol intake, vape use, pre-vaccination SARS-CoV-2 infection status, asthma, atopy, heart disease, arterial disease, kidney disease, major neurological condition, COPD, immunodeficiency, self-assessed general health, vaccine type, quarter of second dose, timing between first and second dose, multivitamin use, vitamin D use, beta-2 adrenergic agonists, statins, ACE inhibitors, inhaled corticosteroids, inhaled bronchodilators, immunosuppressants, SSRIs, anticholinergics, chronic paracetamol use, long covid status

4, adjusted for BMI, age, sex, vigorous exercise, light exercise, highest educational level attained, anxiety/depression, dietary restrictions, pre-vaccination SARS-CoV-2 infection status, asthma, atopy, diabetes, arterial disease, heart disease, major neurological condition, immunodeficiency, self-assessed general health, vaccine type, quarter of first dose, timing of first dose, multivitamin use, vitamin D use, beta-2 adrenergic agonists, statins, proton pump inhibitors, inhaled corticosteroids, inhaled bronchodilators, SSRIs, non- SSRIs, thiazides, SGLT2 inhibitors, anti-platelet drugs, aspirin use, BCG vaccination, long covid status

5, adjusted for age, sex, highest educational level attained, light exercise, anxiety/depression, alcohol intake, dietary restrictions, pre-vaccination SARS-CoV-2 infection status, asthma, atopy, arterial disease, major neurological condition, cancer, immunodeficiency, self-assessed general health, vaccine type, quarter of second dose, multivitamin use, beta-2 adrenergic agonists, statins, proton pump inhibitors, H2-receptor antagonists, inhaled corticosteroids, inhaled bronchodilators, SSRIs, anticholinergics, anti-platelet drugs, sex hormone therapy, aspirin use, paracetamol use long covid status

# **Supplementary Figures**

## **Supplementary Figure 1.** Participant Flow


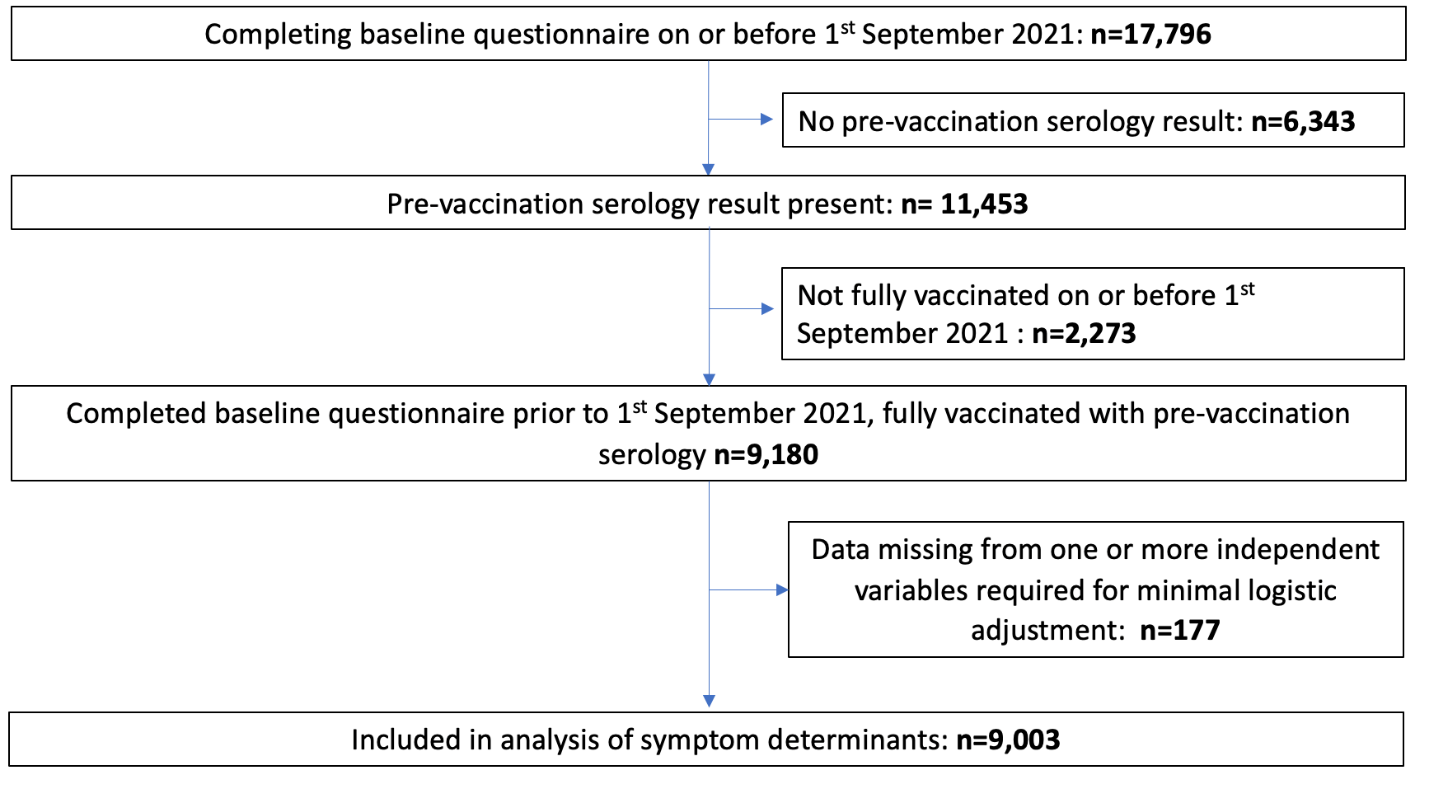


## **Supplementary Figure 2.** Pre-vaccination anti-S titres in participants who experienced symptomatic vs. asymptomatic SARS-CoV-2 infection
