# Supplementary material for: Incidence determinants and serological correlates of reactive symptoms following SARS-CoV-2 vaccination
Source: NPJ Vaccines. 2023 Feb 25;8:26. doi: 10.1038/s41541-023-00614-0 (PMC9959934; doi:10.1038/s41541-023-00614-0)
Supplement: Supplementary file 2 — REPORTING SUMMARY [file 41541_2023_614_MOESM2_ESM.pdf]

nature portfolio

Corresponding author(s): 02214R

Last updated by author(s): Sep 12, 2022

# Reporting Summary

Nature Portfolio wishes to improve the reproducibility of the work that we publish. This form provides structure for consistency and transparency in reporting. For further information on Nature Portfolio policies, see our Editorial Policies and the Editorial Policy Checklist.

Please do not complete any field with "not applicable" or n/a. Refer to the help text for what text to use if an item is not relevant to your study.
For final submission: please carefully check your responses for accuracy; you will not be able to make changes later.

## Statistics

For all statistical analyses, confirm that the following items are present in the figure legend, table legend, main text, or Methods section.

| n/a | Confirmed | |
|---|---|---|
| ☐ | ☒ | The exact sample size ($n$) for each experimental group/condition, given as a discrete number and unit of measurement |
| ☐ | ☒ | A statement on whether measurements were taken from distinct samples or whether the same sample was measured repeatedly |
| ☐ | ☒ | The statistical test(s) used AND whether they are one- or two-sided<br>*Only common tests should be described solely by name; describe more complex techniques in the Methods section.* |
| ☐ | ☒ | A description of all covariates tested |
| ☐ | ☒ | A description of any assumptions or corrections, such as tests of normality and adjustment for multiple comparisons |
| ☐ | ☒ | A full description of the statistical parameters including central tendency (e.g. means) or other basic estimates (e.g. regression coefficient) AND variation (e.g. standard deviation) or associated estimates of uncertainty (e.g. confidence intervals) |
| ☐ | ☒ | For null hypothesis testing, the test statistic (e.g. $F$, $t$, $r$) with confidence intervals, effect sizes, degrees of freedom and $P$ value noted<br>*Give P values as exact values whenever suitable.* |
| ☒ | ☐ | For Bayesian analysis, information on the choice of priors and Markov chain Monte Carlo settings |
| ☒ | ☐ | For hierarchical and complex designs, identification of the appropriate level for tests and full reporting of outcomes |
| ☐ | ☒ | Estimates of effect sizes (e.g. Cohen's $d$, Pearson's $r$), indicating how they were calculated |

*Our web collection on statistics for biologists contains articles on many of the points above.*

## Software and code

Policy information about availability of computer code

| | |
|---|---|
| Data collection | Qualtrics |
| Data analysis | Stata |

For manuscripts utilizing custom algorithms or software that are central to the research but not yet described in published literature, software must be made available to editors and reviewers. We strongly encourage code deposition in a community repository (e.g. GitHub). See the Nature Portfolio guidelines for submitting code & software for further information.

## Data

Policy information about availability of data

All manuscripts must include a data availability statement. This statement should provide the following information, where applicable:

- Accession codes, unique identifiers, or web links for publicly available datasets
- A description of any restrictions on data availability
- For clinical datasets or third party data, please ensure that the statement adheres to our policy

The data that support the findings of this study are available from the corresponding author upon reasonable request.

## Human research participants

Policy information about studies involving human research participants and Sex and Gender in Research.

| | |
|---|---|
| Reporting on sex and gender | We reported data on sex at birth, self reported. We present the sex distribution in our population. The questionnaire was completed by participants in English, where we used standard terminology for collecting such information. We do present associations between sex as a part of a logistic multivariate model. These are presented in Table 1, participant flow is presented in Figure 1. |
| Population characteristics | The mean age of those contributing data to analyses was 61.3 years (range 16.6–88.0), 70.7% were female, 96.4% identified their ethnic origin as White, and 88.4% were living in England. |
| Recruitment | Participants were invited via a national media campaign. |
| Ethics oversight | Leicester South Research Ethics Committee (ref 20/EM/0117) |

Note that full information on the approval of the study protocol must also be provided in the manuscript.

# Field-specific reporting

Please select the one below that is the best fit for your research. If you are not sure, read the appropriate sections before making your selection.

☒ Life sciences ☐ Behavioural & social sciences ☐ Ecological, evolutionary & environmental sciences

# Life sciences study design

All studies must disclose on these points even when the disclosure is negative.

| | |
|---|---|
| Sample size | We estimated that a minimum sample size of 10,964 would be required to detect a difference of at least 2% in the proportion of exposed vs. unexposed participants experiencing a given binary outcome [equivalent to an odds ratio (OR) of 1.08], with 90% power, for a binary exposure with maximum variability (probability 0.50 changing to 0.52) and a moderate correlation (R2 = 0.4) with other variables in a logistic regression model, using a two-sided test and 5% significance. This post-vaccination symptom analysis was a pragmatic study including all participants meeting the inclusion criteria, with no sample size specified. |
| Data exclusions | We excluded those who had no pre-vaccination serology (n=6,343) or who had not completed a full vaccination regimen (n=2,273). We also excluded those with missing data for independent variables required for minimal adjustment in the regression models (n=177). |
| Replication | Not applicable to an observational, real-world study. |
| Randomization | Not relevant because the study design is observational. |
| Blinding | Study participants were unaware of hypotheses being tested and reports of symptoms were given before antibody test results were fed back to them. |

# Reporting for specific materials, systems and methods

We require information from authors about some types of materials, experimental systems and methods used in many studies. Here, indicate whether each material, system or method listed is relevant to your study. If you are not sure if a list item applies to your research, read the appropriate section before selecting a response.

## Materials & experimental systems

| n/a | Involved in the study | |
|---|---|---|
| ☒ | ☐ | Antibodies |
| ☒ | ☐ | Eukaryotic cell lines |
| ☒ | ☐ | Palaeontology and archaeology |
| ☒ | ☐ | Animals and other organisms |
| ☐ | ☒ | Clinical data |
| ☒ | ☐ | Dual use research of concern |

## Methods

| n/a | Involved in the study | |
|---|---|---|
| ☒ | ☐ | ChIP-seq |
| ☒ | ☐ | Flow cytometry |
| ☒ | ☐ | MRI-based neuroimaging |

## Clinical data

Policy information about clinical studies

All manuscripts should comply with the ICMJE guidelines for publication of clinical research and a completed CONSORT checklist must be included with all submissions.

| | |
|---|---|
| Clinical trial registration | NCT04330599 |
| Study protocol | Available at https://clinicaltrials.gov/ct2/show/NCT04330599 |
| Data collection | The study was nested within COVIDENCE UK, which is a prospective longitudinal population-based observational study of coronavirus disease in the UK population (www.qmul.ac.uk/covidence). The study was launched on 1st May 2020, and closed to enrolment on 6th October 2021. This paper reports findings from analyses of data collected up to 1st September 2021. |
| Outcomes | Predefined outcomes were specified in study protocol. |





This checklist template is licensed under a Creative Commons Attribution 4.0 International License, which permits use, sharing, adaptation, distribution and reproduction in any medium or format, as long as you give appropriate credit to the original author(s) and the source, provide a link to the Creative Commons license, and indicate if changes were made. The images or other third party material in this article are included in the article's Creative Commons license, unless indicated otherwise in a credit line to the material. If material is not included in the article's Creative Commons license and your intended use is not permitted by statutory regulation or exceeds the permitted use, you will need to obtain permission directly from the copyright holder. To view a copy of this license, visit http://creativecommons.org/licenses/by/4.0/
